# Multi-Source, Fault-Tolerant, and Robust Navigation Method for Tightly Coupled GNSS/5G/IMU System

**DOI:** 10.3390/s25030965

**Published:** 2025-02-05

**Authors:** Zhongliang Deng, Zhichao Zhang, Zhenke Ding, Bingxun Liu

**Affiliations:** School of Electronic Engineering, Beijing University of Posts and Telecommunications, Beijing 100876, China

**Keywords:** trusted PNT services, fault tolerance navigation, fault detection and troubleshooting, distributed fusion, robust navigation

## Abstract

The global navigation satellite system (GNSS) struggles to deliver the precision and reliability required for positioning, navigation, and timing (PNT) services in environments with severe interference. Fifth-generation (5G) cellular networks, with their low latency, high bandwidth, and large capacity, offer a robust communication infrastructure, enabling 5G base stations (BSs) to extend coverage into regions where traditional GNSSs face significant challenges. However, frequent multi-sensor faults, including missing alarm thresholds, uncontrolled error accumulation, and delayed warnings, hinder the adaptability of navigation systems to the dynamic multi-source information of complex scenarios. This study introduces an advanced, tightly coupled GNSS/5G/IMU integration framework designed for distributed PNT systems, providing all-source fault detection with weighted, robust adaptive filtering. A weighted, robust adaptive filter (MCC-WRAF), grounded in the maximum correntropy criterion, was developed to suppress fault propagation, relax Gaussian noise constraints, and improve the efficiency of observational weight distribution in multi-source fusion scenarios. Moreover, we derived the intrinsic relationships of filtering innovations within wireless measurement models and proposed a time-sequential, observation-driven full-source FDE and sensor recovery validation strategy. This approach employs a sliding window which expands innovation vectors temporally based on source encoding, enabling real-time validation of isolated faulty sensors and adaptive adjustment of observational data in integrated navigation solutions. Additionally, a covariance-optimal, inflation-based integrity protection mechanism was introduced, offering rigorous evaluations of distributed PNT service availability. The experimental validation was carried out in a typical outdoor scenario, and the results highlight the proposed method’s ability to mitigate undetected fault impacts, improve detection sensitivity, and significantly reduce alarm response times across step, ramp, and multi-fault mixed scenarios. Additionally, the dynamic positioning accuracy of the fusion navigation system improved to 0.83 m (1σ). Compared with standard Kalman filtering (EKF) and advanced multi-rate Kalman filtering (MRAKF), the proposed algorithm achieved 28.3% and 53.1% improvements in its 1σ error, respectively, significantly enhancing the accuracy and reliability of the multi-source fusion navigation system.

## 1. Introduction

The competition for resilient positioning, navigation, and timing (PNT) is a strategic area which directly impacts national security [1]. With the rapid evolution of mobile communication networks and artificial intelligence, the widespread adoption of smart devices has become crucial to the growth of location-based services (LBSs) [2]. Many redundant PNT sensors are embedded in hidden application scenarios and platforms, such as smart spatiotemporal networks and the next-generation industrial internet. The core challenge remains to provide high-precision, continuous and reliable navigation services to users in highly dynamic and complex environments.

The Global Navigation Satellite System (GNSS) serves as the most critical solution for LBS, offering high accuracy, real-time capabilities, and extensive coverage, thereby providing continuous and stable navigation information to users in open outdoor environments [3]. However, in urban canyons, bridges, and tunnels, GNSS signals suffer from significant degradation due to nonline-of-sight and multipath effects, which severely constrain positioning accuracy and continuity. To address these GNSS limitations, multi-sensor fusion approaches have been investigated, incorporating technologies such as inertial navigation systems (IMUs), LiDAR SLAM, UWB, Bluetooth, and axle speed sensors [3]. Li [4] proposed a method employing graph optimization tightly integrated with an IMU, which in comparison with innovation Kalman filtering (KF) on simulated data demonstrated an ability to effectively identify satellite faults, even under GNSS reliability contamination. The IMU, as a standalone navigation system, offers independent positioning solutions and anti-jamming capabilities but is susceptible to considerable positional error drift during GNSS signal interruptions [5]. To minimize the risk of positioning failure, Zhen et al. [6,7] demonstrated the complementary nature of LiDAR and ultra wideband (UWB) sensors in geometrically degraded environments, such as elongated narrow spaces, achieving robust localization estimates via probabilistic sensor fusion methods.

As next-generation communication technology, the fifth generation (5G) has already achieved extensive indoor and outdoor coverage globally. With its low latency, high bandwidth, and broad coverage, 5G has the potential to become critical technology for addressing the limitations of GNSS signals. In the 3rd Generation Partnership Project (3GPP) Rel-16 [8] standard, 5G positioning capabilities are specified to achieve an accuracy of 3 m (at 80%) indoors and 10 m (at 80%) outdoors, with end-to-end latency of less than 1 second. As the Rel-18 [9] version progresses—marking the first release of 5G-Advanced—the positioning accuracy of 5G is expected to reach centimeter-level precision, with applications expanding to autonomous driving, vehicle-to-everything (V2X), and other side link scenarios. China Telecom Guangdong led the nation’s first commercial implementation of high-precision indoor positioning using a cost-effective 5G UTDOA 1 point X solution [10], significantly reducing the costs associated with achieving high-precision positioning through 5G cellular networks. Regarding GNSS/5G/IMU integrated positioning [11], Yin et al. [12] introduced an adaptive integrated navigation approach based on Kalman filtering, integrating a GNSS, 5G, and an IMU to address frequent satellite signal lock losses and non-line-of-sight errors in seamless indoor-outdoor environments. However, the lack of robust estimation techniques results in degradation of the positioning performance under high-interference conditions. It is worth noting that while technologies such as WiFi-RTT and Bluetooth can provide some RTT measurements over short distances or in indoor environments, 5G solutions, relying on a standardized, large-bandwidth, low-latency, and wide-coverage network foundation, are able to provide superior positioning capabilities over a wider range with higher accuracy and in more complex scenarios.

In practical applications, navigation systems are subject to inevitable errors and faults due to human interference, adverse environments, and hardware aging [13]. If not identified and immediately excluded, these issues can lead to significant degradation in positioning accuracy or even severe errors, which is unacceptable for safety-critical navigation systems [14]. Thus, effective fault detection and exclusion (FDE) schemes are essential for maintaining the reliability and precision of integrated navigation systems. The concept of integrity monitoring for navigation systems originates in the safety domain [15,16], representing a confidence metric in the correctness of position estimates, including the ability to alert users when reliability is compromised. Early FDE approaches primarily focused on redundant navigation sources within global navigation satellite systems (GNSSs), with the aim of detecting anomalies in satellite measurements, receiver hardware, or signal distortions. A widely adopted approach is receiver autonomous integrity monitoring (RAIM) [17,18,19]. In recent years, scholars have explored a variety of fault detection and exclusion (FDE) methods to perform integrity monitoring, which can be roughly classified into position domain and measurement domain approaches, depending on whether fault detection (FD) is derived from observations or positioning solutions [20]. In the position-domain approach, representative FDE algorithms include multiple solution separation (MSS), multiple hypothesis solution separation (MHSS), and advanced receiver autonomous integrity monitoring (ARAIM) [21]. These algorithms make decisions by comparing the test statistics of full-set and subset solutions. Furthermore, Chen [22] proposed a two-level integrity monitoring strategy for multi-source information fusion navigation, removing fault features at both the system and sensor levels and being applicable to integrated navigation systems like GNSSs, 5G, and barometers (BAs). Inspired by the excellent performance of MHSS ARAIM, researchers have started applying MHSS in Kalman filter integrity monitoring (KFIM) [23]. MHSS has been shown to often yield lower protection levels (PLs) than traditional methods [23], though its high computational cost limits further development of this method. A representative measurement-domain approach is autonomous integrity monitoring based on extrapolation (AIME) [24], which uses deformed residual vectors from the filtering process as fault detection statistics. Building upon this foundation, Yang [25] developed an IMU/GNSS integrated navigation system for urban environments featuring fault detection and exclusion (FDE) capabilities but did not consider the possibility of erroneous IMU measurements. Kaddour [26] proposed a multi-fault detection algorithm using in-formation space observation projection, which involves differentiating the state vectors in a Kalman filter (KF) [27]. Nevertheless, the rapid variability of reference satellites in urban canyons diminished the algorithm’s efficacy. Another issue in GNSS/5G/IMU integrated systems is the presence of undetected measurement faults. Lee [27] employed snapshot innovations for fault detection, yet this method exhibited poor sensitivity to ramp faults. Furthermore, undetected faults tend to accumulate, causing inaccuracies in the filter innovation sequence during recursive computations [28] and resulting in an error-tracking phenomenon which weakens fault detection performance.

To ensure the provision of precise, continuous, and reliable positioning, navigation, and timing (PNT) services, we present a fault-tolerant and robust navigation approach designed for resilient integration of GNSS, 5G, and IMU systems. Sub-filters achieve a tight coupling of observation data, while the main filter dynamically allocates information according to the quality of the observation residuals. A weighted robust adaptive filter based on the maximum correlation entropy criterion is derived, effectively addressing issues of nonpositivity or singularity in the iterative noise covariance estimation process and eliminating error-tracking phenomena. Furthermore, an improved AIME method, termed TSAIME, is developed to mitigate performance degradation caused by variations in the number of signals between epochs in sequential methods [29]. A covariance-optimal, expansion-based integrity crossing protection strategy is proposed, and the optimal scalar expansion strategy yields a conservative estimate of the diagonal matrix crossing. Through fault exclusion and sensor recovery verification, the dynamically adjusted fusion model is better suited to meet the requirements of resilient PNT fusion navigation under complex positioning scenarios.

This paper initially presents the tightly integrated GNSS/5G/IMU framework and its corresponding observation model. The causes of error tracking are then explained in detail, followed by an in-depth elaboration of the construction of the weighted robust adaptive filter. A comprehensive scheme for fault detection, exclusion, and sensor recovery verification is subsequently proposed, including the computation of protection levels and an evaluation of algorithm availability. Lastly, the field test results are analyzed to assess the performance of the proposed method when subjected to various fault types, and conclusions are drawn accordingly.

## 2. System Framework and System Modeling

This section begins by presenting the architecture of the GNSS/5G/IMU integrated navigation system and provides a high-precision, seamless positioning solution for indoor and outdoor environments. The measurement transformation models for the GNSS, 5G, and the IMU are explained in detail.

### 2.1. System Framework

**Figure 1:**
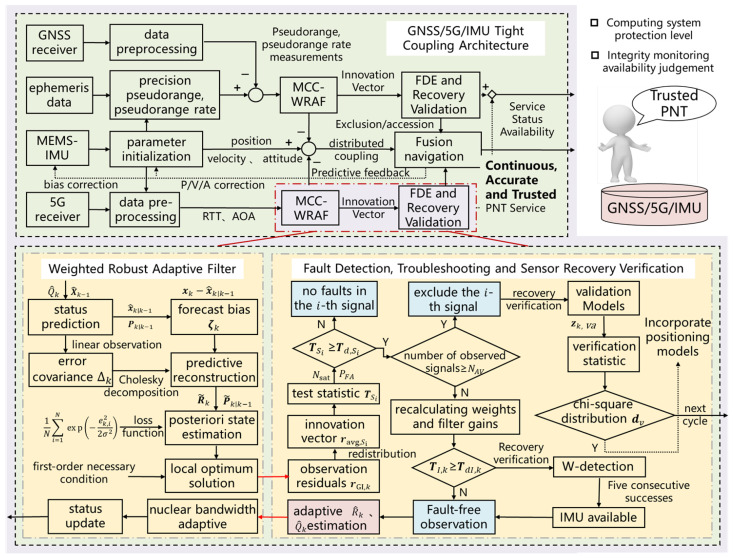
A weighted robust adaptive filtering and all-source fault detection framework for GNSS/5G/IMU tightly coupled navigation.

The system framework is illustrated in Figure 1, comprising three main components: tightly coupled GNSS/5G/IMU integration, robust weighted filtering based on the maximum correlation entropy criterion, and fault detection, exclusion, and sensor recovery verification. The IMU serves as a reference navigation source, initializing the generation of navigation solutions which include the position, velocity, and attitude, which are subsequently fused with GNSS and 5G data to form independent sub-filters. The GNSS utilizes the differences between the IMU-predicted pseudoranges and pseudorange rates as the measurement vector, while 5G processes the observed round-trip time (RTT) and angle of arrival (AOA) similarly to the IMU. The MCC is integrated into the WRAK estimation framework, where posterior residuals are monitored to ensure that the noise matrix remains positive definite and symmetric, deriving the noise covariance update process in non-Gaussian environments. Dynamic corrections are applied to innovation vectors and covariance matrices to compute test statistics, which are compared against corresponding detection thresholds to sequentially verify wireless signal and IMU measurement states, identifying faulty navigation sources and adjusting fault exclusion strategies based on the number of available signals. Additionally, to facilitate the prompt restoration of unreliable sensors, the verification model periodically collects measurement residuals from faulty sensors, constructs test statistics, and assesses sensor performance, reintegrating compliant sensors back into the navigation solution. Following the MCC-WRAF update, the estimated state error vector is employed to correct the IMU-generated navigation solution and adjust biases, yielding an integrated navigation result. Lastly, an integrity risk tree is constructed to compute system protection levels, assess the availability of the navigation state, and deliver continuous, precise, and reliable PNT services to end users.

### 2.2. GNSS/5G/IMU Tightly Coupled Integration Model

**Figure 2:**
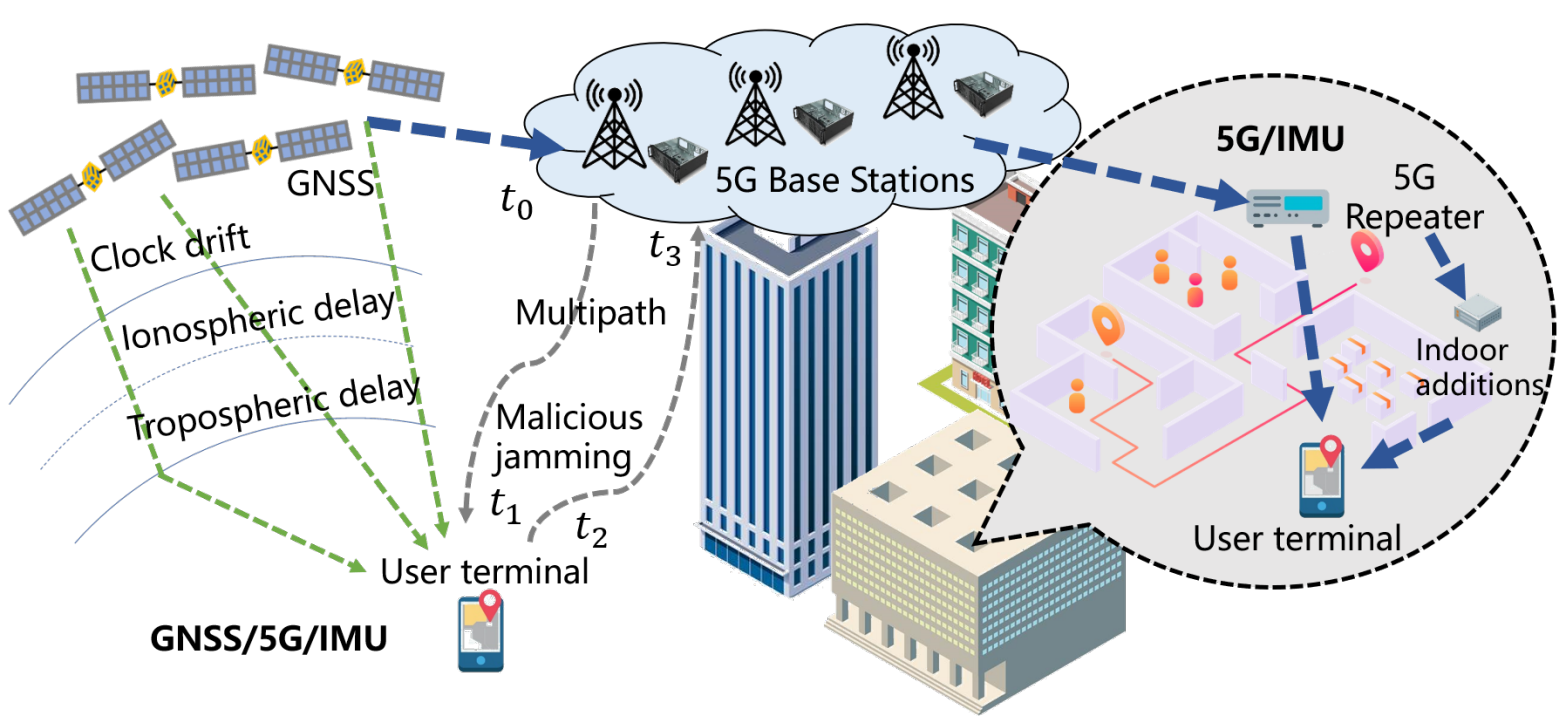
GNSS/5G/IMU seamless and continuous indoor and outdoor positioning solution.

Due to the effects of multipath and satellite visibility obstructions, GNSS receivers are susceptible to significant errors. The incorporation of 5G mitigates challenges such as rapid fluctuations in outdoor signal sources and indoor positioning blind spots, while the IMU contributes autonomous, continuous, real-time, and high-resilience capabilities against interference. Figure 2 illustrates a GNSS/5G/IMU integrated solution which offers seamless and continuous positioning for both indoor and outdoor environments. Due to the lack of compliant 5G real test environments, this paper focuses on outdoor testing and verification.

Based on different sensor combinations, the system is divided into two components: tightly coupled GNSS/IMU integration and tightly coupled 5G/IMU integration. The local optimal solutions derived from the sub-filters are fused to obtain the final navigation result. GNSS typically operates in the Earth-centered, Earth-fixed (ECEF) coordinate system, whereas 5G positioning generally operates in a station-centered coordinate system. In this paper, all state vectors are unified and converted into the ECEF frame to establish the dynamic model of the inertial control system. Typically, the IMU has a higher output frequency compared with the GNSS or 5G. Thus, timestamps from the GNSS and 5G are aligned, and the lower output frequency is used for time synchronization during the fusion process. Detailed sub-filter construction is described in Section 3, and the global state estimation at epoch *k* for the system can be represented by:

(1) $$\begin{array}{c} P_{g,k}={\left(\sum_{i=1}^{2}W_{i}\cdot P_{i,k}^{-1}\right)}^{-1}\\ Q_{g,k}={\left(\sum_{i=1}^{2}W_{i}\cdot Q_{i,k}^{-1}\right)}^{-1}\\ {\hat{X}}_{g,k}=P_{g,k}\sum_{i=1}^{2}\left(W_{i}\cdot P_{i,k}^{-1}{\hat{X}}_{i,k}\right) \end{array}$$

where $P_{i,k}$ represents the state error covariance matrix for node *i* at epoch *k*, $Q_{i,k}$ denotes the process noise covariance matrix for node *i* at epoch *k*, and ${\hat{X}}_{i,k}$ indicates the local state estimate for node *i*, to which a specific weight is assigned:

(2) $$W_{i}=\frac{P_{i}^{-1}}{\sum_{i=1}^{2}P_{i}^{-1}}$$

where $P_{i}^{-1}$ represents the inverse of the covariance matrix for node *i*. The main filter performs only the time update step, and the global estimation results are fed back to the sub-filters based on a specific information allocation criterion [30]:

(3) $$\begin{array}{c} {\hat{X}}_{i,k}={\hat{X}}_{g,k}\\ P_{i,k}=\beta_{i}^{-1}P_{g,k}\\ Q_{i,k}=\beta_{i}^{-1}Q_{g,k},i=1,2 \end{array}$$

where $\beta_{i}$ represents the information distribution coefficient, which satisfies $\beta_{1}+\beta_{2}=1$. To improve the consistency of the feedback, a covariance consistency constraint is introduced to guarantee the convergence of each sub-filter’s covariance:

(4) $$\min_{P_{i,k}}\sum_{i}{∥P_{i,k}-\beta_{i}^{-1}P_{g,k}∥}^{2}+\lambda \cdot h\left(P_{i,k}\right)$$

In this equation, $h(P_{i,k})$ represents the consistency constraint condition, and λ is the Lagrange multiplier.

The tightly coupled structure necessitates a complex signal tracking loop design, whereas the loosely coupled architecture relies heavily on the IMU, resulting in imprecise positioning when wireless signals experience faults. In contrast to the aforementioned schemes, the tightly coupled approach offers a more viable solution under resource constraints, balancing efficiency and performance. The state and measurement equations of the sub-filter can be expressed as follows:

(5) $$\left\{\begin{array}{c} x_{k}=F_{k-1}x_{k-1}+w_{k-1}\\ z_{k}=h_{k}\left(x_{k}\right)+v_{k} \end{array}\right.$$

In this equation, $w_{k}$ and $v_{k}$ denote mutually uncorrelated process noise and measurement noise, respectively. The function $h_{k}\left(\right)$ represents the nonlinear measurement function, and $F_{k-1}$ is the nonlinear state transition function. The state error vector of the system at epoch *k* can be represented as follows:

(6) $$\delta x_{k}=\left[{\left(\delta x_{G,k}\right)}^{T}{\left(\delta x_{I,k}\right)}^{T}{\left(\delta x_{5G,k}\right)}^{T}\right]$$

Further unfolding yields

(7) $$\begin{array}{c} \delta x_{G,k}={\left[\delta b_{G}\cdots \delta d_{G}\right]}^{T}\\ \delta x_{I,k}={\left[{\left(\delta p_{I,k}^{e}\right)}^{T}{\left(\delta v_{I,k}^{e}\right)}^{T}{\left(\delta \varphi_{I,k}^{e}\right)}^{T}\varepsilon^{T}\cdot \gamma^{T}\right]}^{T}\\ \delta x_{5G,k}={\left[\delta b_{5G}\cdots \delta d_{5G}\right]}^{T} \end{array}$$

In this equation, $\delta p_{I,k}^{e}$, $\delta v_{I,k}^{e}$, and $\delta \phi_{I,k}^{e}$ represent the position, velocity, and attitude error vectors in the ECEF coordinate frame, respectively, while ϵ and γ denote the gyroscope and accelerometer errors, respectively, both of which are assumed to follow a first-order Gauss–Markov process, and $\delta b_{i}$ and $\delta d_{i}$ correspond to the receiver clock bias and clock drift, respectively.

### 2.3. GNSS Measurement Model

The measurement vector comprises pseudorange observations and pseudorange rate observations from the GNSS and IMU. A pseudorange observation is defined as the difference between the satellite-derived pseudorange and the predicted pseudorange, which is calculated from the distance between the satellite navigation solution and the inertial navigation position. The pseudorange rate observation is calculated in a similar manner. The measurement vector in the discrete-time domain is denoted by

(8) $$z_{k}^{GNSS}=\left[\begin{array}{c} \rho_{GNSS}-\rho_{INS}\\ {\dot{\rho }}_{GNSS}-{\dot{\rho }}_{INS} \end{array}\right]=H_{t,k}^{GNSS}\left[\begin{array}{c} \delta x_{G,k}\\ \delta x_{I,k} \end{array}\right]+\varepsilon_{t,k}^{GNSS}$$

where $\rho_{GNSS}$ and ${\dot{\rho }}_{GNSS}$ represent the pseudorange measurement and rate vectors of the GNSS receiver, respectively, and $\rho_{INS}$ and ${\dot{\rho }}_{INS}$ are the corresponding predicted values from the INS. The measurement matrix is $H_{t,k}^{GNSS}$, and the noise vector $\varepsilon_{t,k}^{GNSS}$ follows a Gaussian white noise model with zero mean and the covariance $R_{t,k}^{GNSS}$.

The satellite pseudorange measurement and the predicted pseudorange are expressed as follows:

(9) $$\rho_{GNSS,m}=\rho_{R,m}+c\delta t+T+\varepsilon$$

(10) $$\rho_{R,m}=\sqrt{{\left(x^{R}-d_{sx}^{m}\right)}^{2}+{\left(y^{R}-d_{sy}^{m}\right)}^{2}+{\left(z^{R}-d_{sz}^{m}\right)}^{2}}$$

(11) $$\rho_{INS,m}=\sqrt{{\left(x^{INS}-d_{sx}^{m}\right)}^{2}+{\left(y^{INS}-d_{sy}^{m}\right)}^{2}+{\left(z^{INS}-d_{sz}^{m}\right)}^{2}}$$

where $\rho_{GNSS,m}$ represents the measured pseudorange from the *m*th satellite to the receiver, cδt accounts for the distance error due to receiver and satellite clock bias, *T* denotes the ionospheric and tropospheric errors, and ϵ includes the residual biases. Here, $\rho_{R,m}$ is the true distance between the receiver and the satellite, $\left(x^{R},y^{R},z^{R}\right)$ represents the receiver’s position, $\left(d_{sx}^{m},d_{sy}^{m},d_{sz}^{m}\right)$ is the *m*th satellite’s position, and $\left(x^{INS},y^{INS},z^{INS}\right)$ is the position estimated by the IMU.

The satellite pseudorange rate and predicted pseudorange rate are computed as follows:

(12) $${\dot{\rho }}_{GNSS,m}=\left[\left(x^{R}-d_{sx}^{m}\right)\left(v_{x}^{R}-dv_{sx}^{m}\right)+\left(y^{R}-d_{sy}^{m}\right)\left(v_{y}^{R}-dv_{sy}^{m}\right)+\left(z^{R}-d_{sz}^{m}\right)\left(v_{z}^{R}-dv_{sz}^{m}\right)\right]/\rho_{R,m}+\delta^{GNSS}+v_{\rho ,GNSS}$$

(13) $${\dot{\rho }}_{INS,m}=\left[\left(x^{INS}-d_{sx}^{m}\right)\left(v_{x}^{INS}-dv_{sx}^{m}\right)+\left(y^{INS}-d_{sy}^{m}\right)\left(v_{y}^{INS}-dv_{sy}^{m}\right)+\left(z^{INS}-d_{sz}^{m}\right)\left(v_{z}^{INS}-dv_{sz}^{m}\right)\right]/\rho_{R,m}+\delta^{INS}+v_{\rho ,INS}$$

where $\left(v_{x}^{R},v_{y}^{R},v_{z}^{R}\right)$ denotes the receiver’s velocity, $\left(dv_{sx}^{m},dv_{sy}^{m},dv_{sz}^{m}\right)$ indicates the velocity of the *m*th satellite, and $\left(v_{x}^{INS},v_{y}^{INS},v_{z}^{INS}\right)$ represents the velocity predicted by the IMU, while $\delta^{\mathrm{GNSS}\;}$ and $\delta^{INS}$ are the drift of the clock frequency and $v_{\rho ,GNSS}$ and $v_{\rho ,INS}$ account for the unknown error.

By performing a first-order Taylor expansion on $\rho_{GNSS,m}$ and $\rho_{INS,m}$, we obtain the following:

(14) $$\rho_{GNSS,m}-\rho_{INS,m}=\frac{x^{R}-d_{sx}^{m}}{∥x^{R}-d_{sx}^{m}∥}\left(x^{P}-x^{R}\right)+\frac{y^{R}-d_{sy}^{m}}{∥y^{R}-d_{sy}^{m}∥}\left(y^{P}-y^{R}\right)+\frac{z^{R}-d_{sz}^{m}}{∥z^{R}-d_{sz}^{m}∥}\left(z^{P}-z^{R}\right)-c\delta t+\varepsilon$$

Here, $\left(x^{P},y^{P},z^{P}\right)$ denotes the estimated receiver position, and let $\left(\Delta x,\Delta y,\Delta z\right)=\left(x^{P}-x^{R},y^{P}-y^{R},z^{P}-z^{R}\right)$. We then have

(15) $$\rho_{GNSS}-\rho_{INS}=\left[\begin{array}{c} \rho_{GNSS,1}-\rho_{INS,1}\\ \rho_{GNSS,1}-\rho_{INS,2}\\ \vdots \\ \rho_{GNSS,N}-\rho_{INS,N} \end{array}\right]={\left[\begin{array}{ccc} e_{x,1} & e_{y,1} & e_{z,1}\\ e_{x,2} & e_{y,2} & e_{z,2}\\ \vdots & \vdots & \vdots \\ e_{x,N} & e_{y,N} & e_{z,N} \end{array}\right]}_{N\times 3}{\left[\begin{array}{c} \Delta x\\ \Delta y\\ \Delta z \end{array}\right]}_{3\times 1}-{\left[\begin{array}{c} c\delta t\\ c\delta t\\ \vdots \\ c\delta t \end{array}\right]}_{N\times 1}+{\left[\begin{array}{c} \varepsilon_{1}\\ \varepsilon_{2}\\ \vdots \\ \varepsilon_{N} \end{array}\right]}_{N\times 1}$$

where $e_{x,m}=\frac{x^{R}-d_{sx}^{m}}{∥x^{R}-d_{sx}^{m}∥}$, $e_{y,m}=\frac{y^{R}-d_{sy}^{m}}{∥y^{R}-d_{sy}^{m}∥}$, and $e_{z,m}=\frac{z^{R}-d_{sZ}^{m}}{∥z^{R}-d_{sz}^{m}∥}$. *N* denotes the number of observable satellites. Similarly, for the pseudorange rate, we can remodel it as follows:

(16) $${\dot{\rho }}_{GNSS,m}=e_{x,m}\left(v_{x}^{R}-dv_{sx}^{m}\right)+e_{y,m}\left(v_{y}^{R}-dv_{sy}^{m}\right)+e_{z,m}\left(v_{z}^{R}-dv_{sz}^{m}\right)+\delta^{GNSS}+v_{\rho ,GNSS}$$

(17) $${\dot{\rho }}_{INS,m}=e_{x,m}\left(v_{x}^{INS}-dv_{sx}^{m}\right)+e_{y,m}\left(v_{y}^{INS}-dv_{sy}^{m}\right)+e_{z,m}\left(v_{z}^{INS}-dv_{sz}^{m}\right)+\delta^{INS}+v_{\rho ,INS}$$

Furthermore, we can obtain

(18) $${\dot{\rho }}_{GNSS}-{\dot{\rho }}_{INS}=\left[\begin{array}{c} {\dot{\rho }}_{GNSS,1}-{\dot{\rho }}_{INS,1}\\ {\dot{\rho }}_{GNSS,2}-{\dot{\rho }}_{INS,2}\\ \vdots \\ {\dot{\rho }}_{GNSS,N}-{\dot{\rho }}_{INS,N} \end{array}\right]={\left[\begin{array}{ccc} e_{x,1} & e_{y,1} & e_{z,1}\\ e_{x,2} & e_{y,2} & e_{z,2}\\ \vdots & \vdots & \vdots \\ e_{x,N}\cdot & e_{y,N}\cdot & e_{z,N} \end{array}\right]}_{N\times 3}{\left[\begin{array}{c} \Delta v_{x}\\ \Delta v_{y}\\ \Delta v_{z} \end{array}\right]}_{3\times 1}-{\left[\begin{array}{c} c\delta \\ c\delta \\ \vdots \\ c\delta \end{array}\right]}_{N\times 1}+{\left[\begin{array}{c} v_{\dot{\rho }}^{1}\\ v_{\dot{\rho }}^{2}\\ \vdots \\ v_{\dot{\rho }}^{N} \end{array}\right]}_{N\times 1}$$

### 2.4. 5G Measurement Model

The 5G observation vector comprises the RTT and AOA. Multi-RTT, defined in 3GPP Rel-16 [8], is a 5G measurement method which, compared with TOA ranging, uses the round-trip time of uplink and downlink signals to mitigate synchronization errors between the base station and the terminal. The formula is given as follows:

(19) $$T_{RTT}=\frac{\left(t_{1}-t_{0}\right)+\left(t_{3}-t_{2}\right)}{2}=\frac{\left(t_{3}-t_{0}\right)-\left(t_{2}-t_{1}\right)}{2}$$

where $t_{0}$ and $t_{3}$ represent the times when the 5G base station sends and receives signals, respectively, while $t_{1}$ and $t_{2}$ denote the times when the terminal receives and sends signals, respectively. Since both are relative time differences measured against their own clocks, no synchronization is required between the base station and terminal during the observation process.

Given that standard-compliant macrocell coverage is still incomplete, incorporating angle measurements is crucial for single-base-station positioning. The angle of arrival (AOA) is calculated on the 5G base station side, relying on its massive array antenna. The angle measurements are broadcast from the base station, enabling terminal-side positioning. The complete observation equation at epoch *k* is

(20) $$z_{k}^{5G}=\left[\begin{array}{c} \rho_{5G}-\rho_{INS}\\ \phi_{5G}-\phi_{INS} \end{array}\right]=\left[\begin{array}{c} RTT_{1,k}^{5G}-RTT_{1,k}^{INS}\\ \vdots \\ RTT_{n,k}^{5G}-RTT_{n,k}^{INS}\\ Azi_{1,k}^{5G}-Azi_{1,k}^{INS}\\ \vdots \\ Azi_{n,k}^{5G}-Azi_{n,k}^{INS}\\ Ele_{1,k}^{5G}-Ele_{1,k}^{INS}\\ \vdots \\ Ele_{n,k}^{5G}-Ele_{n,k}^{INS} \end{array}\right]=H_{t,k}^{5G}\left[\begin{array}{c} \delta x_{5G,k}\\ \delta x_{I,k} \end{array}\right]+\varepsilon_{t,k}^{5G}$$

where $\rho_{5G}$ and $\phi_{5G}$ denote the RTT and AOA measurements from the receiver, respectively, while $\phi_{INS}$ is the IMU-projected angle observation at time *k*. $RTT_{n,k}^{5G}$, $Azi_{n,k}^{5G}$, and $Ele_{n,k}^{5G}$ correspond to the RTT, azimuth, and elevation of the *n*th 5G base station at time *k*, respectively. $RTT_{n,k}^{INS}$, $Azi_{n,k}^{INS}$, and $Ele_{n,k}^{INS}$ are the RTT, azimuth, and elevation as projected by the IMU at time *k* for the *n*th base station, respectively. The term $\varepsilon_{t,k}^{5G}$ contains RTT and AOA noise. Furthermore, we have

(21) $$\rho_{5G}=\left[\begin{array}{c} cT_{RTT}\\ \theta \\ \varphi \end{array}\right]=\left[\begin{array}{c} \sqrt{{\left(x_{e}-e_{0}\right)}^{2}+{\left(y_{e}-n_{0}\right)}^{2}+{\left(z_{e}-u_{0}\right)}^{2}}\\ \arctan\frac{x_{e}-e_{0}}{y_{e}-n_{0}}\\ \arctan\frac{\left(z_{e}-u_{0}\right)}{\sqrt{{\left(x_{e}-e_{0}\right)}^{2}+{\left(y_{e}-n_{0}\right)}^{2}}} \end{array}\right]$$

where θ and ϕ denote the azimuth and elevation angles, respectively, and ${\left(e_{0},n_{0},u_{0}\right)}^{T}$ and ${\left(x_{e},y_{e},z_{e}\right)}^{T}$ represent the positions of the 5G base station and receiver in the ENU coordinate system, respectively, with transformations from the ECEF coordinate system as follows:

(22) $$\left[\begin{array}{c} x_{e}\\ y_{e}\\ z_{e} \end{array}\right]=\left[\begin{array}{ccc} -\sin\left(\lambda_{L}\right) & \cos\left(\lambda_{L}\right) & 0\\ -\sin\left(\phi_{L}\right)\cos\left(\lambda_{L}\right) & -\sin\left(\phi_{L}\right)\sin\left(\lambda_{L}\right) & \cos\left(\phi_{L}\right)\\ \cos\left(\phi_{L}\right)\cos\left(\lambda_{L}\right) & \cos\left(\phi_{L}\right)\sin\left(\lambda_{L}\right) & \sin\left(\phi_{L}\right) \end{array}\right]\left[\begin{array}{c} x^{R}\\ y^{R}\\ z^{R} \end{array}\right]$$

where $\lambda_{L}$ and $\phi_{L}$ represent the longitude and latitude of the location, respectively. Given that the observation equation involves both the distance and angle, and particularly when the AOA observation equation exhibits a strong nonlinear relationship between the angle and distance, directly deriving the first-order Jacobian is complicated and may result in accuracy loss. We followed the approach of Jiao et al. [31], which converts arc observations obtained from inverse trigonometric functions into distance-related expressions and then linearizes them. The solution process is similar to that in Section 2.3, yielding the expression for the measurement relationship matrix $H_{t,k}^{5G}$:

(23) $$H_{t,k}^{5G}=\left[\begin{array}{c} 0_{1\times 3}\;0_{1\times 3}\;E_{1,k}^{RTT}\;0_{1\times 3}\;0_{1\times 3}\\ \vdots \\ 0_{1\times 3}\;0_{1\times 3}\;E_{n,k}^{RTT}\;0_{1\times 3}\;0_{1\times 3}\\ 0_{1\times 3}\;0_{1\times 3}\;E_{1,k}^{Azi}\;0_{1\times 3}\;0_{1\times 3}\\ \vdots \\ 0_{1\times 3}\;0_{1\times 3}\;E_{n,k}^{Azi}\;0_{1\times 3}\;0_{1\times 3}\\ 0_{1\times 3}\;0_{1\times 3}\;E_{1,k}^{Ele}\;0_{1\times 3}\;0_{1\times 3}\\ \vdots \\ 0_{1\times 3}\;0_{1\times 3}\;E_{n,k}^{Ele}\;0_{1\times 3}\;0_{1\times 3} \end{array}\right]$$

where

(24) $$E_{n,k}^{RTT}={\left[\begin{array}{c} \frac{\partial RTT_{n,k}^{INS}}{\partial x}\\ \frac{\partial RTT_{n,k}^{INS}}{\partial y}\\ \frac{\partial RTT_{n,k}^{INS}}{\partial z} \end{array}\right]}^{T},E_{n,k}^{Azi}={\left[\begin{array}{c} \frac{\partial Azi_{n,k}^{INS}}{\partial x}\\ \frac{\partial Azi_{n,k}^{INS}}{\partial y}\\ 0 \end{array}\right]}^{T},E_{n,k}^{Ele}={\left[\begin{array}{c} \frac{\partial Ele_{n,k}^{INS}}{\partial x}\\ \frac{\partial Ele_{n,k}^{INS}}{\partial y}\\ \frac{\partial Ele_{n,k}^{INS}}{\partial z} \end{array}\right]}^{T}$$

## 3. Weighted Robust Adaptive Filter

This section introduces an enhanced weighted robust adaptive filter, building on the Sage-Husa Adaptive Kalman Filter (SHAKF). It begins by explaining the causes of error tracking, followed by a discussion on the fundamentals of maximum correntropy. The MCC is then incorporated into the MRAF framework, leading to the derivation of a maximum correntropy-based adaptive filter.

### 3.1. Error Tracking Phenomenon

During the estimation process, minor faults which do not significantly impact positioning are often undetectable by conventional algorithms. Error tracking describes the cumulative effect of these undetected minor faults, resulting in inaccuracies in test statistics. The detailed analysis is as follows.

Assume the observation at epoch *k* includes faults:

(25) $$z_{k}^{f}=z_{k}+\Delta z_{k}$$

The innovation vector containing the faults can be expressed as

(26) $$r_{k}^{f}=z_{k}^{f}-H_{k}{\hat{x}}_{k|k-1}=r_{k}+\Delta z_{k}$$

where ${\hat{x}}_{k|k-1}$ denotes the state of a prior estimate at moment *k*. The state estimate contains the faults

(27) $${\hat{x}}_{k}^{f}={\hat{x}}_{k|k-1}+K_{k}r_{k}^{f}={\hat{x}}_{k}+K_{k}\Delta z_{k}$$

where $K_{k}$ denotes the filter gain. For the next epoch, the predicted state with unknown faults is

(28) $${\hat{x}}_{k+1|k}^{f}=F_{k+1|k}{\hat{x}}_{k}^{f}={\hat{x}}_{k+1|k}+F_{k+1|k}K_{k}\Delta z_{k}$$

where $F_{k+1|k}$ is the state transfer matrix.The innovation of the filter at epoch k+1 when faults reoccur is

(29) $$\begin{array}{cc} r_{k+1}^{f} & =z_{k+1}^{f}-H_{k+1}{\hat{x}}_{k+1|k}^{f}\\ \; & =z_{k+1}+\Delta z_{k+1}-H_{k+1}{\hat{x}}_{k+1|k}-H_{k+1}F_{k+1|k}K_{k}\Delta z_{k}\\ \; & =r_{k+1}+\Delta z_{k+1}-H_{k+1}F_{k+1|k}K_{k}\Delta z_{k} \end{array}$$

where $\left(-H_{k+1}F_{k+1|k}K_{k}\Delta z_{k}\right)$ reflects the effect of previously undetected faults on the current innovation and $\left(r_{k+1}+\Delta z_{k+1}\right)$ represents the expected impact of current faults on the detection statistic. The presence of the error term leads to a gradual decrease in filter innovation due to error accumulation, which reduces the algorithm’s sensitivity and prevents the detection of new faults.

Thus, a new filtering approach is required to mitigate the impact of accumulated errors on fault detection statistics in the absence of new faults.

### 3.2. Maximum Correlation Entropy Criterion

For the random variables *X* and *Y*, the goal is to maximize their correlation and determine their optimal joint distribution under given constraints. The correlation coefficient us

(30) $$I\left(X,Y\right)=\;\int \int \;K_{X}\left(x\right)K_{Y}\left(y\right)p\left(x,y\right)dxdy$$

Here, $K\left(x,y\right)=K_{X}\left(x\right)K_{Y}\left(y\right)$ represents the kernel function in the feature space obtained through kernel mapping. The Gaussian kernel is commonly used, and it is defined as follows:

(31) $$K\left(x,y\right)=\exp\left(-\frac{{∥x-y∥}^{2}}{2\sigma^{2}}\right)$$

where σ represents the kernel bandwidth, determining the weight distribution between second-order and higher-order moments. The kernel function maps points *x* and *y* from the original space to ϕ(x) and ϕ(y) in a high-dimensional space. Here, ϕ() denotes the mapping function $R^{n}\to H$, where H is the high-dimensional feature space. Consider the Taylor series expansion

(32) $$I\left(X,Y\right)=\sum_{n=0}^{\infty }\frac{{\left(-1\right)}^{n}}{2^{n}\sigma^{2n}n!}G\left[{\left(X-Y\right)}^{2n}\right]$$

where $\left[{\left(-1\right)}^{n}/2^{n}\sigma^{2n}n!\right]$ represents the coefficient of the extended weighting matrix. Unlike the minimum mean square error (MMSE) criterion used in the extended Kalman filter (EKF), unscented Kalman filter (UKF), and their variants, managing non-Gaussian noise through higher-order mapping is especially crucial.

By incorporating Lagrange multipliers and normalization constraints, the objective function can be reformulated as follows:

(33) $$L\left(p,\lambda \right)=-\;\int \int \;p\left(x,y\right)\log p\left(x,y\right)dxdy+\lambda_{1}\left(\;\int \int \;p(x,y)dxdy-1\right)$$

When x(i) is close to y(i), this indicates higher similarity between the two variables, implying a stronger dependency between the samples and resulting in higher entropy. The final optimization problem is expressed as follows:

(34) $$\max_{p(x,y)}\;\int \int \;K_{X}\left(x\right)K_{Y}\left(y\right)p\left(x,y\right)dxdy$$

We seek the optimal solution within the feasible set of possible joint distributions of (X,Y), aiming to make the filtering result approximate the desired signal as closely as possible. A higher correlation coefficient indicates a better probability combination, offering an improved filter solution for non-Gaussian errors.

### 3.3. Weighted Robust Filtering Method Based on Maximum Correlation Entropy Criterion

In dynamic scenarios, Kalman filtering relies on accurate prior state and covariance matrix estimates to balance the error between consecutive observations, ensuring faster convergence. Process and observation noise matrices are usually derived from empirical models, adjusted for the characteristics of the measurement equipment and user expertise, and treated as fixed constants. However, in navigation systems utilizing GNSSs, 5G, and IMUs, constant prior information may not accurately reflect observation quality.

Building on the EKF, the SHAKF enhances adaptability in dynamic systems by estimating the process noise covariance *Q* and observation noise covariance *R* in real time, addressing unpredictable noise characteristics. However, the SHAKF still adheres to the MMSE criterion, resulting in poor performance in non-Gaussian or nonlinear noise conditions. Furthermore, the covariance matrix generated by the SHAKF may lose positive definiteness, causing instability during iteration.

To address the aforementioned constraints, the MMSE criterion is replaced by the MCC criterion, which leverages higher-order statistics to better capture non-Gaussian noise and complex error distributions. An innovative weighted iterative KF and posterior residual estimation are used to ensure the observation noise matrix *R* remains symmetrical and stable throughout the calculation. In the prediction phase, the state transition model and prior state information are used to generate the predicted state at epoch *k*, along with its associated error covariance matrix, given by

(35) $${\hat{x}}_{k|k-1}=F_{k}{\hat{x}}_{k-1|k-1}+B_{k}u_{k}$$

(36) $$P_{k|k-1}=F_{k}P_{k-1|k-1}F_{k}^{T}+Q_{k-1}$$

where $B_{k}$ denotes the control input matrix, $u_{k}$ is the control input vector, and $Q_{k-1}$ is the process noise covariance matrix. Its posterior distribution satisfies $p\left(x_{k}|z_{k}\right)\propto p\left(z_{k}|x_{k}\right)p\left(x_{k}\right)$. The state prediction bias at this moment is

(37) $$\Delta x_{k}=x_{k}-{\hat{x}}_{k|k-1}$$

This is derived from the linear regression between the predicted values and observations:

(38) $$\alpha_{k}=\left[\begin{array}{c} {\hat{x}}_{k|k-1}\\ z_{k} \end{array}\right]-\left[\begin{array}{c} I\\ H_{k} \end{array}\right]x_{k}=\left[\begin{array}{c} -\Delta x_{k}\\ v_{k} \end{array}\right]$$

The state error covariance matrix $P_{k}$ and the observation error covariance matrix are

(39) $$P_{k}=E\left[\left({\hat{x}}_{k}-x_{k}\right){\left({\hat{x}}_{k}-x_{k}\right)}^{T}\right]$$

(40) $$R_{k}=E\left[\left(z_{k}-H_{k}{\hat{x}}_{k}\right){\left(z_{k}-H_{k}{\hat{x}}_{k}\right)}^{T}\right]$$

We can derive the extended error covariance matrix as follows:

(41) $$\begin{array}{cc} \Delta_{k} & \left.=E\left[\begin{array}{c} \Delta x_{k}\\ v_{k} \end{array}\right]{\left[\begin{array}{c} \Delta x_{k}\\ v_{k} \end{array}\right]}^{T}\right]=\left[\begin{array}{cc} P_{k|k-1} & P_{k|k-1}H_{k}^{T}\\ H_{k}P_{k|k-1} & H_{k}P_{k|k-1}H_{k}^{T}+R_{k} \end{array}\right]\\ \; & =\left[\begin{array}{cc} L_{P,k|k-1} & 0\\ 0 & L_{R,k} \end{array}\right]\left[\begin{array}{cc} L_{P,k|k-1}^{T} & 0\\ 0 & L_{R,k}^{T} \end{array}\right]=L_{k}L_{k}^{T} \end{array}$$

where $L_{P,k|k-1}$ and $L_{R,k}$ are the lower triangular matrices of the $P_{k|k-1}$ and $R_{k}$ Cholesky decompositions, respectively. By introducing maximum correntropy as the loss function, the state estimation solution is derived:

(42) $$x_{k}=\arg\max\sum_{i=1}^{m+n}K_{\sigma }\left(\delta_{k}\left(i\right)\right)$$

Here, *m* and *n* denote the dimensions of the state and observation vectors, respectively. The error vector $\delta_{k}\left(i\right)$ is given by

(43) $$\delta_{k}=L_{k}^{-1}\left[\begin{array}{c} {\hat{x}}_{k|k-1}\\ z_{k} \end{array}\right]-L_{k}^{-1}\left[\begin{array}{c} I\\ H_{k} \end{array}\right]x_{k}=L_{k}^{-1}\left[\begin{array}{c} {\hat{x}}_{k|k-1}-x_{k}\\ z_{k}-H_{k}x_{k} \end{array}\right]=\left[\begin{array}{c} L_{x}\left({\hat{x}}_{k|k-1}-x_{k}\right)\\ L_{z}\left(z_{k}-H_{k}x_{k}\right) \end{array}\right]$$

Furthermore, the optimal solution is obtained:

(44) $$\sum_{i=1}^{m+n}\left[\nabla_{x_{k}}\left(K_{\sigma }\left(\delta_{k}\left(i\right)\right)\delta_{k}\left(i\right)\right)\right]=0$$

State estimation is expressed as $x_{k}=W_{k}^{-1}W_{k}$, where

(45) $$W_{k}=\left[{\hat{x}}_{k|k-1}\cdot z_{k}\right]{\left(L_{k}^{T}\right)}^{-1}\left[\begin{array}{cc} G_{P} & 0\\ 0 & G_{R} \end{array}\right]L_{k}^{-1}\left[\begin{array}{c} {\hat{x}}_{k|k-1}\\ z_{k} \end{array}\right]$$

(46) $$\begin{array}{cc} G_{P} & =\mathrm{diag}\left(G_{\sigma }\left(\delta_{k}\left(i\right)\right),\ldots ,G_{\sigma }\left(\delta_{k}\left(m\right)\right)\right)\\ G_{R} & =\mathrm{diag}\left(G_{\sigma }\left(\delta_{k}\left(i\right)\right),\ldots ,G_{\sigma }\left(\delta_{k}\left(n\right)\right)\right) \end{array}$$

in which diag denotes the diagonal symbol. We can reformulate the predicted error covariance matrix and the measurement noise variance as follows:

(47) $${\tilde{P}}_{k|k-1}=\left(L_{P,k|k-1}\right)I{\left(L_{P,k|k-1}\right)}^{T}$$

(48) $${\tilde{R}}_{k}=L_{R,k}G_{R,k}^{-1}L_{R,k}^{T}$$

In the SHAKF, the observation noise matrix $R_{k}$ frequently becomes non-positive definite during estimation, occasionally resulting in a singular matrix. The high sensitivity of these changes can cause numerical instability and divergence in the filter. Following the approach of Akhlaghi et al. [32], we further obtain

(49) $${\hat{R}}_{k}=\left(1-d_{k}\right){\tilde{R}}_{k}+d_{k}\left(\left(z_{k}-H_{k}{\hat{x}}_{k}\right){\left(z_{k}-H_{k}{\hat{x}}_{k}\right)}^{T}+H_{k}{\tilde{P}}_{k}{H_{k}}^{T}\right)$$

where $d_{k}=\left(1-b\right)/\left(1-b^{k+1}\right)$ represents the adjustment factor and *b* is typically set between 0.95 and 0.99. The process noise covariance matrix $Q_{k}$ is estimated similarly to the observation noise. To avoid matrix singularity while keeping complexity low, it is calculated as follows:

(50) $${\hat{Q}}_{k}=\left(1-d_{k}\right){\hat{Q}}_{k-1}+d_{k}\left(K_{k}e_{k}e_{k}^{T}K_{k}^{T}\right)$$

Here, $e_{k}$ represents the innovation residual. The updated covariance is then incorporated into the measurement update process:

(51) $$\begin{array}{c} {\tilde{K}}_{k}={\tilde{P}}_{k|k-1}{H_{k}}^{T}{\left(H_{k}{\tilde{P}}_{k|k-1}{H_{k}}^{T}+{\hat{R}}_{k}\right)}^{-1}\\ {\hat{x}}_{k}={\hat{x}}_{k|k-1}+{\tilde{K}}_{k}\left(z_{k}-H_{k}{\hat{x}}_{k|k-1}\right)\\ P_{k}=\left(I-{\tilde{K}}_{k}H_{k|k-1}\right){\tilde{P}}_{k|k-1} \end{array}$$

In this equation, ${\tilde{K}}_{k}$, ${\hat{x}}_{k}$, and $P_{k}$ represent the gain matrix, posterior state, and posterior covariance matrix, respectively. The choice of kernel bandwidth σ is critical for filter robustness, as suggested in [33].

The MCC-WRAF method proposed in this section assigns weights according to the reliability of various measurements, suppresses error tracking, and ensures that the detection statistic accurately reflects the actual fault magnitude. In the absence of faults, the corrected measurement noise matrix helps mitigate accumulated errors and enhances positioning accuracy.

## 4. Fault Detection, Exclusion, and Sensor Recovery Verification Scheme

**Figure 3:**
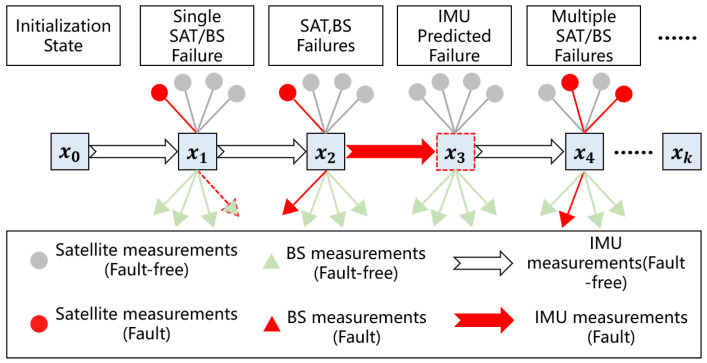
State estimation procedure for different fault modes.

Figure 3 illustrates the state estimation models for various fault modes, followed by a detailed explanation of the comprehensive fault detection and isolation scheme, which performs stepwise identification of fault sources. A fault exclusion strategy is introduced to maintain positioning performance during faults. Next, a sensor recovery verification method is developed to reintegrate reliable sensors into the navigation system. Lastly, a covariance-optimal inflation strategy for integrity boundary protection was developed to rigorously evaluate the reliability of positioning outcomes.

### 4.1. Fault Detection and Separation

When the innovation vector of the MCC-WRAF is affected by faults, it directly influences the positioning results. Inspired by the AIME approach, we developed a novel fault detection and isolation scheme where a sliding window extends over the innovation vector along the time sequence, according to the type and number of information sources.

Taking the GNSS as an example, measurement modeling is used to separate GNSS and IMU faults, allowing for IMU fault detection even when faulty satellites are detected and excluded. The AIME test statistic is $T_{k}=r_{k}^{T}G_{k}^{-1}r_{k}$, where

(52) $$G_{k}^{-1}=\sum_{i=k-l+1}^{k}g_{GNSS,i}^{-1}$$

(53) $$r_{k}=G_{k}\sum_{i=k-l+1}^{k}g_{GNSS,i}^{-1}\cdot r_{GNSS,i}$$

(54) $$r_{GNSS,i}=O_{N\times 3}^{e}\cdot \left(\varepsilon_{I}+b_{I}\right)-\varepsilon_{G}-b_{G}={\left[\begin{array}{ccc} e_{x,1} & e_{y,1} & e_{z,1}\\ e_{x,2} & e_{y,2} & e_{z,2}\\ \vdots & \vdots & \vdots \\ e_{x,N} & e_{y,N} & e_{z,N} \end{array}\right]}_{N\times 3}\cdot \left(\varepsilon_{I}+b_{I}\right)-\varepsilon_{G}-b_{G}$$

(55) $$g_{GNSS,k}=H_{t,k}^{GNSS}{\tilde{P}}_{k|k-1}{\left(H_{t,k}^{GNSS}\right)}^{T}+{\hat{R}}_{k}$$

Here, $\varepsilon_{I}$ and $b_{I}$ denote the noise and fault vectors derived from the IMU, respectively, while $\varepsilon_{G}$ and $b_{G}$ represent the noise and fault vectors derived from the GNSS, respectively. $T_{k}$ follows a chi-squared distribution with degrees of freedom equal to the number of visible satellites, which explains why the detection threshold dynamically changes due to non-line-of-sight interference and obstructions, while $g_{GNSS,k}$ represents the covariance matrix of the innovation.

The detection threshold $T_{d,k}$ is determined by the false alarm rate $P_{FA}$ and the number of satellites:

(56) $$F\left(T_{d,k}\cdot N\right)+P_{\mathrm{FA}}=1$$

Here, F() represents the cumulative distribution function of the central chi-squared distribution, with $P_{\mathrm{FA}}$ typically set to $10^{-5}$. When $T_{k}\geq T_{d,k}$, AIME usually considers that a fault has occurred.

Variations in signal quantity across different epochs directly affect the computation of the innovation vector and the covariance matrix, impacting fault detection performance. Specifically, the rapid transformation of the observation matrix $H_{k}$ leads to instability in $g_{GNSS,k}$, and fluctuations in degrees of freedom alter the chi-squared distribution shape, increasing the risks of false alarms and missed detections. Weighted approaches and sliding windows cannot fully address the performance degradation caused by these variations in sequential methods. To solve this, we propose a temporal sequence AIME (TSAIME) method, which decomposes the innovation vector based on the source number, shifting the detection focus from multiple satellites within the same epoch to multiple epochs for a fixed satellite.

We first compute the test statistics for all visible signals, with the detection statistic for SAT-1 defined as $T_{S1}=r_{S1}^{T}G_{S1}^{-1}r_{S1}$, where

(57) $$\begin{array}{c} G_{S1}^{-1}=\sum_{i=k-l+1}^{k}g_{ki}^{S_{1}-1}\\ r_{S1}=G_{S1}^{-1}\sum_{i=k-l+1}^{k}g_{ki}^{S_{1}-1}r_{ki}^{S_{1}} \end{array}$$

Here, $g_{ki}^{S_{j}}\left(j=1,2\ldots S_{N}\right)$ denotes the covariance matrix of the innovation vector $r_{ki}^{S_{i}}$ at time ki, and $r_{S1}$ is composed of the elements of the innovation vector related to SAT-1 across different times in the sliding window, where $r_{GI,s1}={\left[\begin{array}{ccc} r_{k1}^{S_{1}} & r_{k2}^{S_{1}} & r_{kl}^{S_{1}} \end{array}\right]}^{T}$. The detection threshold $T_{d,S1}$ for $T_{S1}$ is determined jointly by the false alarm rate $P_{FA}$ and the sliding window length.

When integrated with wireless signals, the IMU is generally assumed to be a stable and reliable navigation tool. However, in specific application scenarios (such as prolonged GNSS outages or sensor damage), IMU errors tend to be cumulative and progressive, leading to faults which can significantly impact system performance and increase positioning errors. Our objective is to detect all-source faults.

First, we construct a test statistic solely influenced by GNSS faults, with the corresponding innovation vector given by

(58) $$r_{G,k}=\left(I_{N\times N}-O_{N\times 3}^{e}\cdot A\right)\cdot r_{GNSS,k}$$

where $A={\left(O_{N\times 3}^{e}\cdot O_{N\times 3}^{e}\right)}^{-1}\cdot O_{N\times 3}^{e}$. The covariance matrix is given by $g_{G,k}=u_{G,k}\cdot g_{GNSS,k}\cdot {u_{G,k}}^{T}$, where $u_{G,k}=O_{N\times 3}^{e}\cdot A-I_{N\times N}$. The 5G fault detection method is similar to that of a GNSS and is performed on a per-signal basis.

Next, the innovation for the IMU fault test statistic is

(59) $$r_{I,k}=A\cdot r_{GNSS,i}=\varepsilon_{I}+b_{I}-A\cdot \left(\varepsilon_{G}+b_{G}\right)$$

During GNSS faults, $r_{I,k}$ cannot be directly used to calculate the IMU test statistic. Therefore, GNSS faults are prioritized for exclusion to minimize the effect of $\left(\varepsilon_{G}+b_{G}\right)$ on IMU fault detection. The corresponding covariance matrix is $g_{G,k}=A\cdot g_{GNSS,k}\cdot A^{T}$. The test statistic $T_{I,k}$ is compared with the detection threshold to determine if the IMU has a fault.

### 4.2. Troubleshooting Strategy

The objective of fault exclusion is to maintain the accuracy of the navigation solution and the stability of the integrated system. Given the unique characteristics of GNSSs, IMUs, and 5G, their fault exclusion methods differ. For a single type of wireless signal, when the number of visible sources exceeds four, the system excludes faulty sources from calculations. However, if the number of visible signals is four or fewer, relevant innovations are weighted accordingly. For GNSSs, the weight calculation is as follows:

(60) $$w_{ki}^{GNSS}=\left\{\begin{array}{cc} 1, & \alpha_{s,r_{Gi}}\leq T_{d,Gi}\\ \frac{\alpha_{s,r_{Gi}}}{T_{d,\mathrm{Gi}}}, & \alpha_{s,r_{Gi}}>T_{d,Gi} \end{array}\right.$$

Here, $\alpha_{s,r_{Gi}}={\left(r_{GNSS,kj}/\sigma_{GNSS,k}^{j}\right)}^{2}$, where $r_{GNSS,kj}$ is the *j*th element of $r_{GNSS,i}$, $\sigma_{GNSS,k}^{j}$ represents the normalized variance of $r_{GNSS,kj}$, and $T_{d,Gi}$ is the corresponding detection threshold.

The IMU serves as a reference filter, and its faults can significantly degrade navigation performance. Upon detection of an IMU fault, the covariance matrix ${\hat{Q}}_{k}$ will be redefined as follows:

(61) $$\tilde{Q_{k}}=\left\{\begin{array}{cc} {\hat{Q}}_{k}, & T_{I,k}\leq T_{dl,k}\\ T_{I,k}\cdot {\hat{Q}}_{k}, & T_{I,k}>T_{dl,k} \end{array}\right.$$

It is worth mentioning that when the IMU is damaged or completely unavailable, positioning is achieved by directly integrating the GNSS and 5G, as suggested by Liu et al. [34]. Sensors excluded due to faults are validated through a recovery model, and those meeting the requirements are reintegrated into the positioning solution.

### 4.3. Sensor Recovery Verification Method

When the system includes unreliable sensors, the verification process will be conducted periodically. The GNSS/5G recovery verification model is essentially similar to an innovation-based chi-squared test, where the observation model is composed of measurements from both unreliable and reliable sensors:

(62) $$Z_{k,\;\mathrm{all}\;}=\left[\begin{array}{c} Z_{k,\;\mathrm{normal}\;}\\ Z_{k,\;\mathrm{fault}\;} \end{array}\right]=\left[\begin{array}{c} H_{k,\;\mathrm{normal}\;}\\ H_{k,\;\mathrm{fault}\;} \end{array}\right]x_{k|k-1}+\left[\begin{array}{c} \varepsilon_{k,\;\mathrm{normal}\;}\\ \varepsilon_{k,\;\mathrm{fault}\;} \end{array}\right]$$

Here, $Z_{k,\;\mathrm{normal}\;}$ denotes the observation value from the reliable sensor, while $Z_{k,\;\mathrm{fault}\;}$ represents the observation from the unreliable sensor. The verification test statistic is derived from the innovations of the unreliable sensor over the period $T_{z}$:

(63) $$\begin{array}{cc} r_{k,v} & =z_{k,\;\mathrm{fault}\;}-H_{k,\;\mathrm{fault}\;}{\hat{x}}_{k|k-1}\\ g_{v,k}^{GNSS} & =H_{k,\;\mathrm{fault}\;}{\tilde{P}}_{k|k-1}H_{k,fault}^{T}+{\hat{R}}_{v,k}\\ d_{v} & =\sum_{t=k}^{k+T_{z}}r_{k,v}^{T}{\left(g_{v,k}^{GNSS}\right)}^{-1}r_{k,v} \end{array}$$

In the verification phase, if $d_{v}$ conforms to a chi-squared distribution, then the sensor is deemed reliable and can be reintegrated into the integrated navigation solution. Otherwise, the sensor is still considered faulty and will wait for the next verification cycle.

We apply W detection [35] to verify the recovery of faulty IMUs, where the verification test statistic and detection threshold are given in least squares form as follows:

(64) $$\begin{array}{cc} r_{k,v}^{I} & =\left|\frac{e_{v}^{T}N_{k,v}Y_{k,v}}{\sqrt{e_{v}^{T}N_{k,v}g_{G,k}N_{k,v}e_{v}}}\right|\\ T_{ver} & =\nabla S_{v}\sqrt{e_{v}^{T}N_{k,v}g_{G,k}N_{k,v}e_{v}} \end{array}$$

where:

(65) $$Y_{k,v}=\left[\begin{array}{c} Z_{k,\;\mathrm{fault}\;}\\ X_{k,\;\mathrm{normal}\;} \end{array}\right],C_{k,v}=\left[\begin{array}{c} H_{k,\;\mathrm{fault}\;}\\ I_{k,\;\mathrm{normal}\;} \end{array}\right],N_{k,v}=\left[\begin{array}{cc} R_{k,\;\mathrm{fault}\;} & 0\\ 0 & P_{k,\;\mathrm{normal}\;} \end{array}\right]$$

Here, $e_{v}$ denotes the unit vector, which is one at the IMU measurement and zero elsewhere, and $\nabla S_{v}$ is the critical outlier value determined by the significance level α. If the condition $r_{k,v}^{I}\leq T_{ver}$ is met for five consecutive verifications, then the IMU is considered ready to rejoin the positioning solution.

### 4.4. Covariance Optimal Expansion-Based Completeness Overrun Protection Strategy

Unknown inter-filter correlations can introduce random fluctuations in the protection level estimates of distributed systems, either elevating or reducing the estimates depending on the correlation strengths. The protection level (PL) is a crucial indicator of the reliability and precision of a positioning system. It provides performance assurance by estimating the maximum possible positioning error within a specified confidence level. For instance, the horizontal protection level (HPL) is used, and the equivalent condition for the applicability of the fault detection and exclusion method is HPL⩽HAL, where the right-hand side represents the horizontal alert limit. The calculation of the HPL is as follows:

(66) $$HPL=\max\left(SLOPE_{i}\times \sigma_{i}\right)\times \sqrt{\lambda }+k\cdot \sigma_{\mathrm{major}\;}$$

In this equation, $SLOPE_{i}$ represents the signal characteristic slope, λ is the non-centrality parameter, which depends on the false alarm rate, missed detection rate, and number of visible signals, and $\sigma_{i}$ and $\sigma_{\mathrm{major}\;}$ represent the standard deviations of the observation noise and positional error, respectively. The characteristic slope is further expressed by

(67) $$SLOPE_{k}=\sqrt{\frac{E{\left[{\hat{x}}_{k|k-1}-x_{k}\right]}^{2}}{\lambda_{k}^{2}}}$$

Furthermore, we obtain

(68) $$\varepsilon_{k}={\hat{x}}_{k|k-1}-x_{k}=K_{k}^{\prime }F\varepsilon_{k-1}+K_{k}v_{k}-K_{k}^{\prime }w_{k-1}$$

In this equation, $K_{k}^{\prime }=I-K_{k}H_{k}$, and by taking the expectation of the above equation, where $E\left[w_{k-1}\right]=0$, we obtain

(69) $$E\left[\varepsilon_{k}\right]=K_{k}^{\prime }F_{k}E\left[\varepsilon_{k-1}\right]+K_{k}E\left[v_{k}\right]$$

Assuming that the algorithm is effective and previous faults have been excluded, for the current fault, $E\left[\varepsilon_{k}\right]=K_{k}\Delta z_{k}$. The non-centrality parameter $\lambda_{k}^{2}$ can be represented by $E\left[g_{k}^{T}\right]S_{k}^{-1}E\left[g_{k}\right]$, where

(70) $$E\left[g_{k}\right]=-H_{k}FE\left[\varepsilon_{k-1}\right]+H_{k}E\left[w_{k-1}\right]+E\left[R_{k}\right]$$

The characteristic slope can be reformulated as follows:

(71) $$SLOPE_{k}=\sqrt{\frac{E{\left[\varepsilon_{e,k}\right]}^{2}+E{\left[\varepsilon_{n,k}\right]}^{2}}{E\left[g_{k}^{T}\right]H_{k}^{-1}E\left[g_{k}\right]}}$$

By substituting the above equation into Equation (66), the covariance matrix of each known filter is adjusted by applying an optimal scalar inflation factor:

(72) $$\tilde{R}=\left[\begin{array}{cccc} {\tilde{P}}_{1} & 0 & \cdots & 0\\ 0 & {\tilde{P}}_{2} & \cdots & 0\\ \vdots & \vdots & \ddots & \vdots \\ 0 & 0 & \cdots & {\tilde{P}}_{M} \end{array}\right]=\left[\begin{array}{cccc} \frac{1}{\omega_{1}}P_{1} & 0 & \cdots & 0\\ 0 & \frac{1}{\omega_{2}}P_{2} & \cdots & 0\\ \vdots & \vdots & \ddots & \vdots \\ 0 & 0 & \cdots & \frac{1}{\omega_{M}}P_{M} \end{array}\right]$$

where $\sum_{i=1}^{i=M}\omega_{i}=1$, $0\leq \omega_{i}\leq 1$. The necessary and sufficient conditions to ensure integrity in distributed PNT systems are as follows:

(73) $$P_{HMI}=P\left\{|\epsilon |>\tilde{PL}L_{ob}|H_{0}\right\}P\left\{H_{0}\right\}+P\left\{|\epsilon |>\tilde{PL_{ob}}|H_{1}\right\}P\left\{{\tilde{D}}_{ob}<T|H_{1}\right\}P\left\{H_{1}\right\}<I_{REQ}$$

## 5. Experimental Results and Analysis

### 5.1. Experiment Description

**Figure 4:**
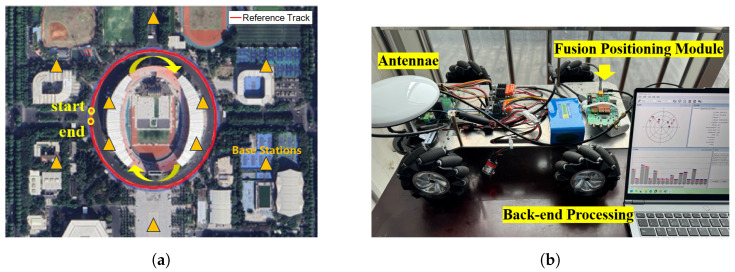
(**a**) Experimental data acquisition path. (**b**) Experimental data acquisition equipment.

**Figure 5:**
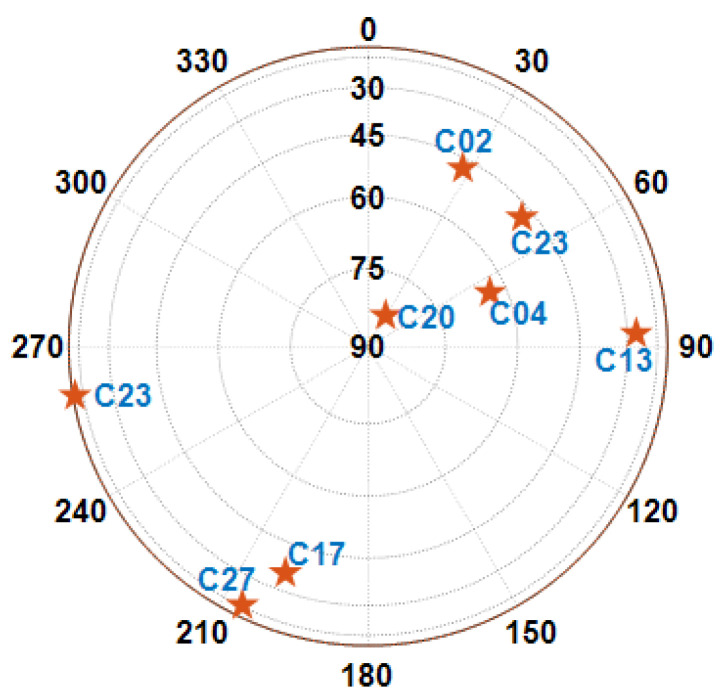
Sky plot showing observable satellites. The pentagrams represent satellites.

A field experiment was conducted in Guangzhou, Guangdong, China to validate the proposed algorithm. The GNSS and IMU used real-world data, and simulated 5G RTT/AOA measurements compliant with 3GPP Rel-16 standards were also used for integrated positioning. The original gyroscope and accelerometer data were collected at a frequency of 100 Hz, while the GNSS measurements were taken at 1 Hz. The 5G simulated terminal was located close to the GNSS antenna phase center, and observation noise was introduced after Gaussian white noise. The 5G RTT and AOA data were synthesized based on the reference trajectories of the carrier and the preset parameters, the standard deviation of the distance measurement was fixed at 1 m, and the standard deviation of the angle measurement from 0 to 5 degrees was in increments of 0.5 degrees. The key parameters shown in Table 1. The simulations used at this stage captured only basic multipath phenomena, and extreme multipath environments such as complex urban canyons need to be studied in depth. The reference true values obtained from the high-precision integrated navigation system typically achieved centimeter-level accuracy, with some segments reaching decimeter-level accuracy. The experiment was carried out bear Tianhe District Stadium in Guangzhou for two loops, and the data collection route and equipment are illustrated in Figure 4. The GNSS module used was NEO-M8L, and the inertial module was ADIS16470. During the operation, the vehicle speed ranged between 1 m/s and 1.5 m/s, with a total data length of 1850 s. The visible satellite map at 1000 s is depicted in Figure 5.

At different times, the navigation sensors’ measurements were affected by faults of different types and magnitudes, which were used to verify the fault detection and exclusion capabilities of our proposed method. Specific fault descriptions are provided in Table 2.

To evaluate the fault detection, exclusion, and sensor recovery verification methods, our proposed solution was compared with AIME, the chi-squared test, and causal subspace fault detection and exclusion (CS-FDE). Furthermore, to validate the effectiveness of the weighted robust adaptive filtering method, comparisons were conducted with weighted least squares (WLS), the EKF, SHAKF, and multi-rate adaptive Kalman filtering (MRAKF). A brief description of each method is given below.

(1) AIME integrates the EKF with innovation vectors to detect outliers in observations, dynamically adjusting the fault detection process based on system states and historical data. It is highly adaptive but lacks real-time responsiveness.
(2) The Chi-squared test uses innovation and covariance matrices to compute statistics and compare them with a threshold, providing strong real-time capability but struggling with complex systems or dynamic changes.
(3) CS-FDE utilizes causality constraints and subspace methods to detect and isolate faults in multi-sensor systems, constructing a fault set related to historical data to capture system dynamics and determine if a fault has occurred.
(4) WLS estimates system weights by minimizing weighted squared errors, performing exceptionally well with varying observation quality but being sensitive to noise covariance matrix settings.
(5) The EKF is an extension of the KF for nonlinear state estimation, characterized by simplicity and robustness, but it relies on accurate prior noise covariance matrix design.
(6) The SHAKF supports dynamic noise covariance adjustment to maintain filtering performance under unknown conditions, albeit with significant computational overhead.
(7) MRAKF addresses discrepancies in state prediction and update intervals in multi-sensor fusion. It weights measurements according to sensor sampling rates, optimizing estimates through multiple measurement integrations.

To evaluate the fault detection accuracy and filtering performance of different methods, we used the root mean square error (RMSE), fault detection rate (FDR), east-north-up (ENU) positioning error, and alarm delay (AD) as performance indicators.

### 5.2. Detection Performance for Step Fault Scenarios

**Figure 6:**
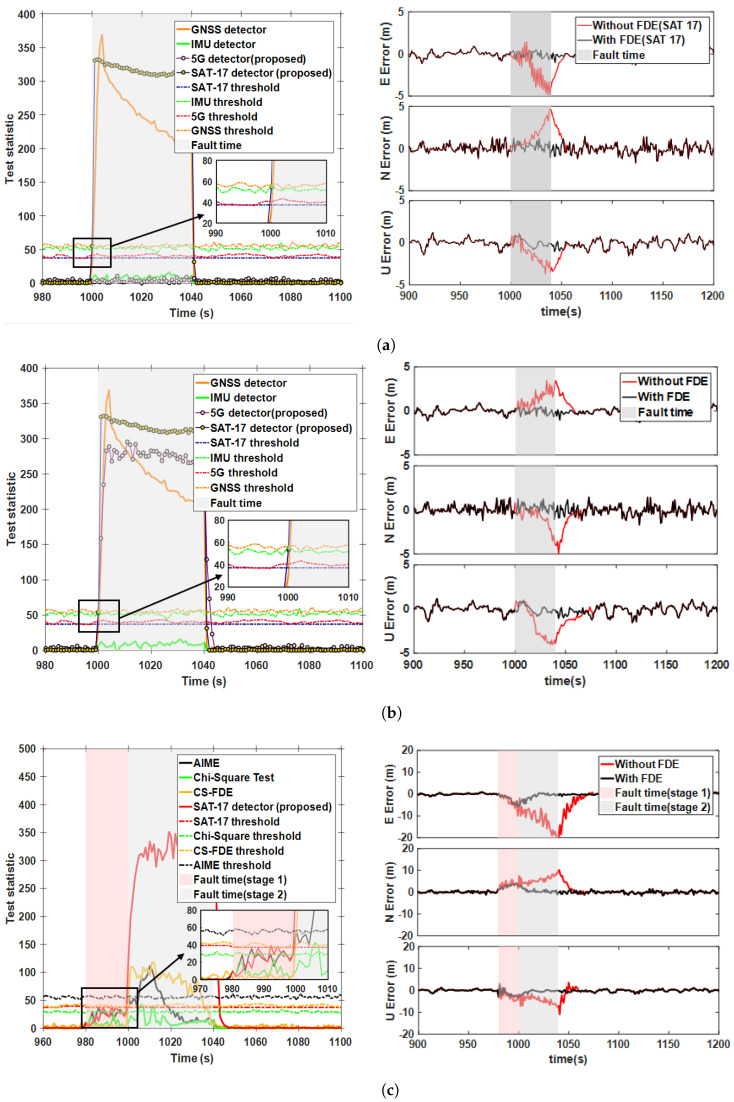
Step fault detection statistic and positioning error curves for GNSS/5G: (**a**) Case 1, (**b**) Case 2, (**c**) Case 4.

Signal clock jumps, severe vibrations, and abnormal IMU temperatures are significant causes of step faults. In this section, Figure 6a compares the detection performance of different methods under a 15 m step fault (case 1 in Table 2). The fault was injected into SAT-17 at the 1000th second and lasted for 41 periods. The results indicate that, compared with the AIME-based GNSS detector, TSAIME also identified the faulty satellite within 3 s, with similar fault detection times for both methods. Additionally, during GNSS fault detection, the test statistics for 5G and the IMU remained below the threshold, further validating the effectiveness of the fault separation method. To further verify the fault exclusion strategy, we compared the positioning errors in the ENU directions before and after FDE. The results show that if faults were not isolated, then the positioning error curve increased, negatively affecting the accuracy of integrated navigation. However, when isolated, the positioning error curve stayed within the normal accuracy limits. It is worth noting that all of our positional error analyses were for sub-filter studies before analyzing the performance of the main filter in a fault-free scenario. After fault detection and exclusion, the position error during faults decreased from a maximum of −4.89 m to −0.69 m, a reduction of over 85%. Figure 6b show the detection and positioning performance after injecting a 15 m step fault into satellites and 5G signals during the same time period (case 2 in Table 2). It can be seen that when the 5G signal included a faulty navigation source, the TSAIME 5G test statistic curve rose sharply and exceeded the threshold quickly without affecting GNSS detection. Table 3 shows the RMSE of the position errors during the fault period. For case 1 and case 2, the 3D RMSE decreased by 78.98% and 75.15%, respectively, after FDE.

**Figure 7:**
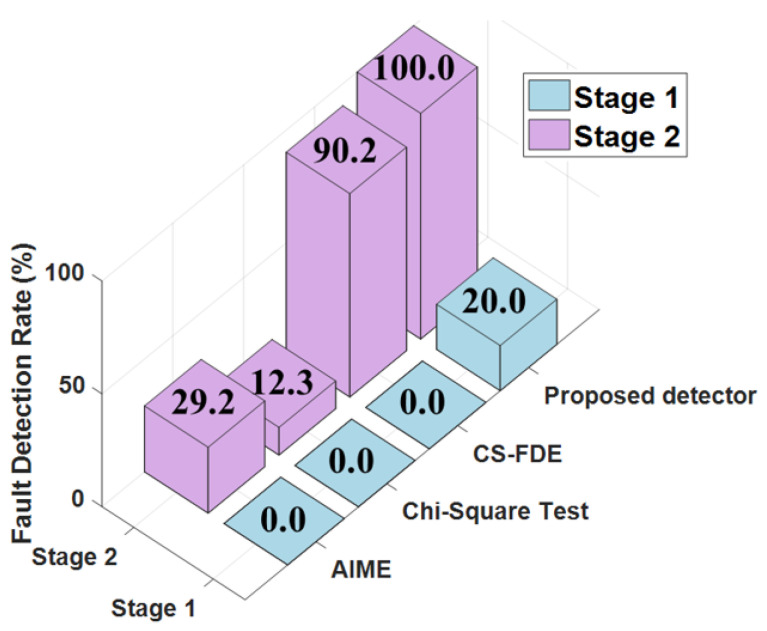
Fault detection rate statistics for the two stages in case 4.

To verify the effectiveness of the proposed filter in eliminating error tracking, the validation in Figure 6c includes two stages (case 4 in Table 2). In the first stage, a 5 m step fault was injected into SAT-17 starting at the 980th second, lasting for 20 periods. In the second stage, a 15 m step fault was injected into the same satellite between 1000 and 1040 s. Figure 7 shows the FDR for case 4 using AIME, the Chi-squared test, the CS-FDE, and the proposed method.

**Figure 8:**
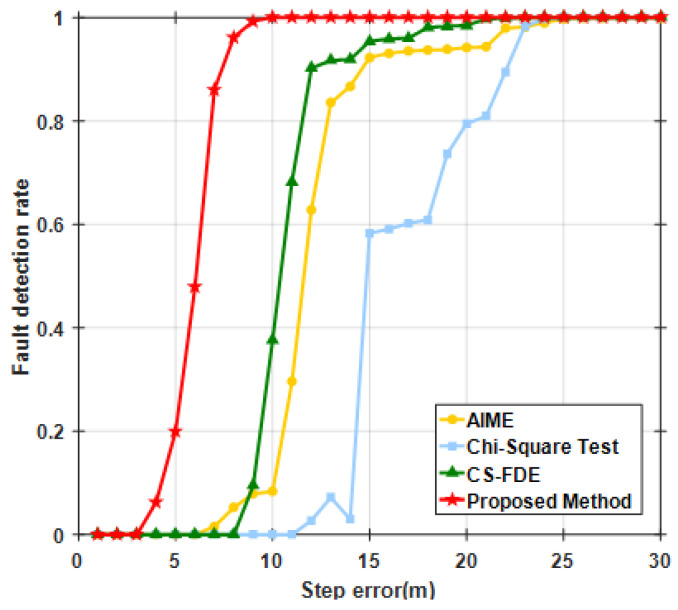
Fault detection rate of SAT-17 under varying step errors.

The results indicate that minor faults in the first stage could not be detected by AIME, the Chi-squared test, or CS-FDE. Due to the single satellite sliding window, the proposed method achieved a detection rate of 20%, reducing the maximum positioning error by 29.3% compared with the case without FDE. In the second stage, the FDR for AIME and the Chi-squared test, which are based on filter innovations, were 29.2% and 12.3%, respectively. This was because accumulated undetected faults led to inaccurate filter innovations, failing to reflect the true changes. The CS-FDE, using historical fault experiences for offline modeling and calculating detection statistics and thresholds based on local outliers, overcame the limitations of innovation filtering during online monitoring, achieving an FDR of 90.2%, which was significantly higher than those of AIME and the Chi-squared test. The adaptive robust estimation method we proposed assigns weights according to observation quality, and even if the contaminated satellite is not detected, the adjusted measurement noise matrix reduces accumulated innovation errors, achieving an FDR of 100%. Compared with no FDE, the maximum positioning error decreased from −22.20 m to −4.76 m, reducing the error by 78.6%. The RMSE decreased by 10.6% in the first stage and by 83.2% in the second stage after FDE. To further assess the detection performance of the proposed method, different step errors were injected into the same satellite during the same time period. Figure 8 compares the fault detection rates of different methods. When the step error was large, all four methods showed good fault detection performance. However, when the step error was less than 15 m, the detection rates of CS-FDE and AIME decreased significantly. Compared with the other three methods, the proposed method was more sensitive to minor faults, achieving 100% detection for a minimum deviation of 10 m.

**Figure 9:**
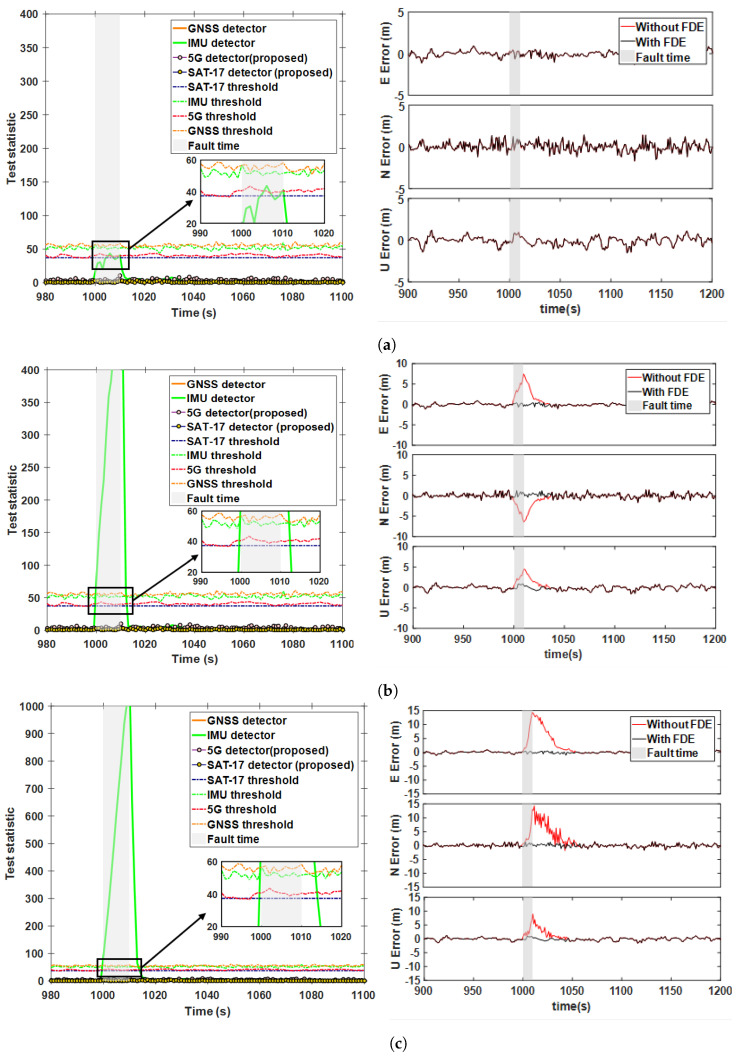
IMU step fault detection statistic and positioning error curves: (**a**) case 3.1, (**b**) case 3.2, and (**c**) case 3.3.

Figure 9 presents the test statistics for IMU faults with different step magnitudes (case 3 in Table 2). In Figure 9a, it is shown that the minor step fault in case 3.1 could not be detected because MCC-WRAF effectively estimated and compensated for small IMU faults in real time, thereby reducing the influence of $\left(\varepsilon_{I}+b_{I}\right)$ on the innovation estimate. This also indicates that IMU errors smaller than one order of magnitude compared with normal operating conditions had negligible effects on the positioning outcomes. Furthermore, Figure 9b,c demonstrate that significant step faults could be promptly detected. Before FDE, the maximum positioning errors for case 3.2 and case 3.3 were 10.8 m and 20.9 m, respectively. After FDE, these maximum errors were reduced by 87.6% and 93.6%, respectively, and the RMSE during the fault period was reduced by 84.9% and 90.5%, respectively, ensuring the operational integrity of the reference source.

### 5.3. Detection Performance for Ramp Fault Scenarios

**Figure 10:**
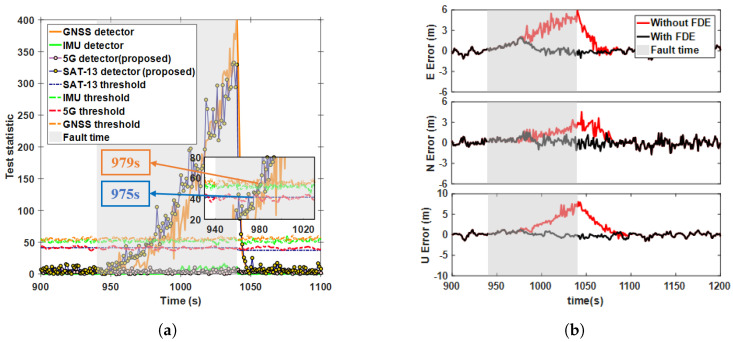
Ramp fault detection statistics and positioning error curves for case 5. (**a**) Comparison of test statistics using different methods. (**b**) East-north-up positioning errors before and after FDE.

**Figure 11:**
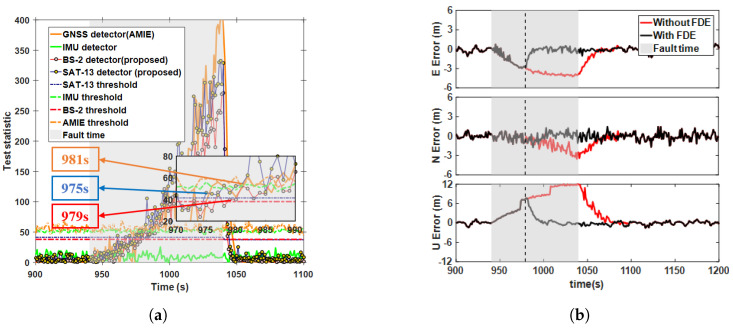
Ramp fault detection statistics and positioning error curves for case 6. (**a**) Comparison of test statistics using different methods. (**b**) East-north-up positioning errors before and after FDE.

**Figure 12:**
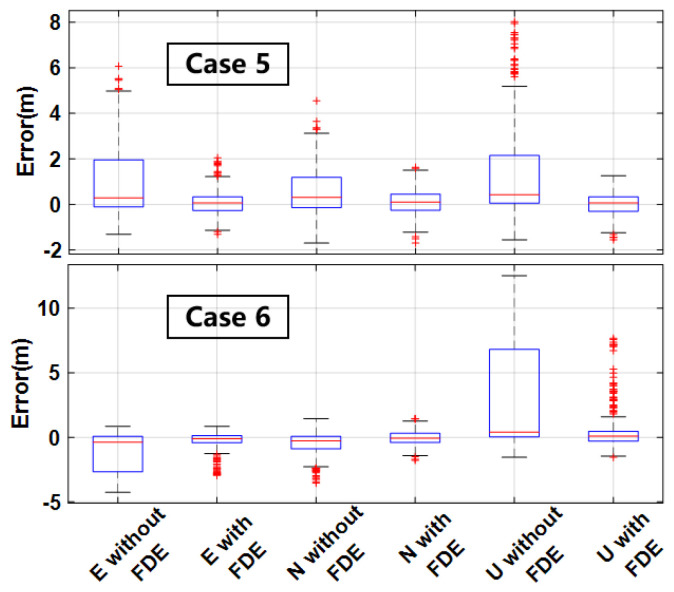
Boxplot distribution of east-north-up positioning errors before and after FDE in ramp fault conditions.

Ramp faults occur due to gradual and continuous deviations in the system or signal, caused by factors such as systematic drift and accumulation of sensor errors. In this subsection, a ramp fault was injected at a rate of 0.1 m / s into SAT-13, lasting 940 to 1040 s. Figure 10a presents a comparison of different test statistics (case 5 in Table 2). The results show that compared to the AIME-based GNSS detector, the proposed method reduced the detection delay by 4 s and improved the alarm efficiency by 10.2%, while the test statistics for 5G and the IMU consistently remained below the threshold. The positioning results in Figure 10b indicate that before the ramp fault was detected, the positioning error also increased due to the fault. Furthermore, the slow-growing fault applied to the sensor clock drift manifested itself primarily in the upward direction. The maximum positioning error decreased from 7.54 m to 1.26 m after FDE, representing a reduction of 83. 2% and the RMSE of the positioning result decreased by 76.1%. Figure 11a illustrates a comparison of different test statistics when a ramp fault of 0.1 m/s was injected simultaneously into SAT-13 and BS-2 for 940–1040 s (case 6 in Table 2). The alarm times for the SAT-13, BS-2 and GNSS detectors were 36 s, 40 s, and 42 s, respectively, demonstrating that the proposed method exhibited superior detection sensitivity compared to the existing methods. In Figure 11b, the maximum positioning error decreased from 11.99 m to 7.67 m after FDE, representing a reduction 36%, and the RMSE of the positioning results decreased by 69.4%, explains the accumulation of outliers in the boxplot in Figure 12 when FDE was applied.

**Figure 13:**
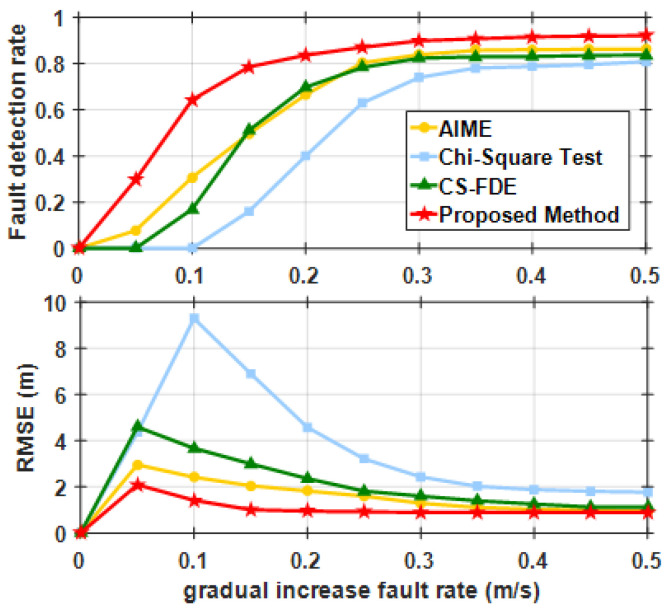
FDRs and RMSEs for various ramp fault conditions.

To further illustrate the fault detection capability and positioning performance under varying ramp fault intensities, ramp faults with rates ranging from 0 to 0.5 m/s were sequentially added to SAT-13 during the same time period. The FDR and RMSE results are presented in Figure 13. Among the methods tested, the Chi-squared test consistently showed the worst performance under all fault intensities. As the ramp fault intensity increased, the fault detection rate also improved, with the proposed method performing better for small ramp faults. However, when the ramp fault rate exceeded 0.3 m/s, the performance of AIME, CS-FDE, and the proposed method converged. The RMSE results indicate that MCC-WRAF effectively reduced the impact of undetected faults during the time from the initiation of the ramp fault to its initial detection.

### 5.4. Detection Performance for Mixed Multi-Fault Scenarios

**Figure 14:**
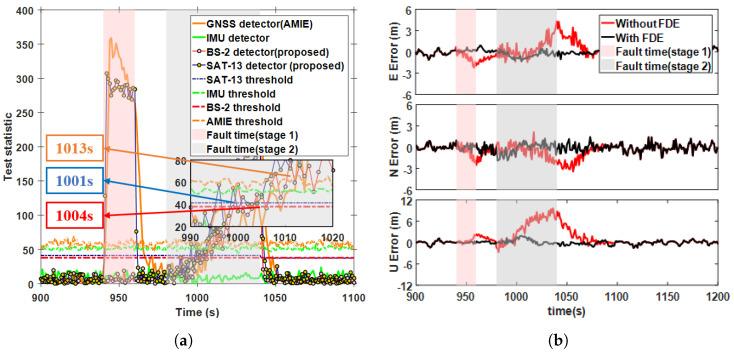
Ramp fault detection statistics and positioning error curves for case 7. (**a**) Comparison of test statistics using different methods. (**b**) East-north-up positioning errors before and after FDE.

To evaluate the performance of the proposed method under multiple fault scenarios, we considered two different fault events which occurred consecutively. In the first stage, a 15 m step fault was injected into the observations of SAT-13 and SAT-20, lasting from 940 to 960 s. In the second stage, a 0.2 m/s ramp fault was added to the SAT-13 and BS-2 measurements, lasting from 980 to 1040 s. Figure 14a illustrates the changes in the detection statistics under the influence of mixed faults. In the first stage, both the AIME-based GNSS detector and the proposed method successfully triggered alarms in 3 s, while the detection statistics for 5G and the IMU remained below the threshold. Notably, the AIME method experienced a delay in detecting the ramp fault during the second stage, influenced by the step fault’s impact on filter innovation during the first stage. The alarm times for the the SAT-13-, BS-2-, and AIME-based GNSS detectors were 22 s, 25 s, and 34 s, respectively. This delay might be because the EKF, when handling the step fault in the first stage, adjusted the gain or noise covariance matrices, reducing the filter’s sensitivity to new faults and thus extending the detection time for the ramp fault. Figure 14b shows the positioning errors before and after FDE during the mult-fault stages. In the first stage, the maximum positioning error was reduced from 2.51 m to 0.99 m after FDE, representing a reduction of 60.3%. In the second stage, the maximum positioning error was reduced from 9.66 m to 1.89 m, an 80.5% reduction. The RMSEs decreased by 69.4% and 77.3% in the first and second stages after FDE, respectively, effectively maintaining positioning performance during multiple fault periods.

**Figure 15:**
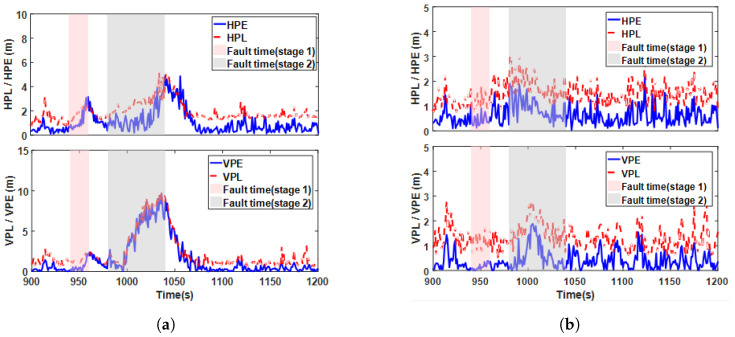
Protection level curves: (**a**) before FDE and (**b**) after FDE.

To further assess the confidence level of the final positioning results, the protection level (PL) was calculated to constrain positioning errors. Using case 7 in Table 2 as an example, the protection level curves of the fusion system before and after fault isolation are shown in Figure 15. The results indicate that during system stability, the horizontal protection level (HPL) and vertical protection level (VPL) were approximately 1.92 m and 1.64 m, respectively. Figure 15a illustrates the results of the calculations before FDE, where the protection level of the system increased significantly when sensor faults occurred, although some epochs may not have been isolated in time. Figure 15b shows the results after FDE, where undetected faults introduced some deviations in the measurements but the protection level and positioning errors remained within the normal range during the fault period.

**Figure 16:**
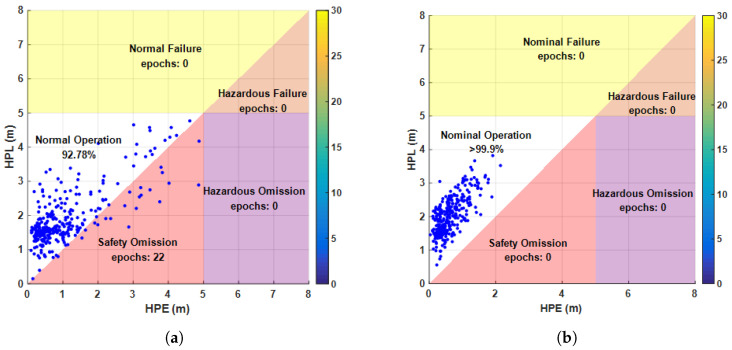
Stanford chart before and after FDE: (**a**) before FDE and (**b**) after FDE.

Referring to high-safety application scenarios such as aircraft approach and landing, engineering surveying, and rail transportation, an HAL of 5 m can provide sufficient horizontal navigation accuracy. Typically, when the positioning error is within the HPL range, and the HPL is less than the alert limit, the solution is considered reliable. Once the positioning error exceeds the HPL, the position estimate becomes unreliable. Based on the magnitudes of the HPE, HPL, and HAL, the position status of the system is further identified in Figure 16. Between 900 and 1200 s, before FDE, due to faults not being eliminated in time, the HPE gradually increased and exceeded the HPL but did not exceed the HAL, indicating unsafe epochs, with the system in a normal state 92.78% of the time. After FDE, the faults were isolated, and the system remained in a normal state for more than 99.9% of the time, demonstrating that the proposed method can provide reliable positioning results under multiple fault disturbances.

### 5.5. Positioning Performance Under Fault-Free Conditions

**Figure 17:**
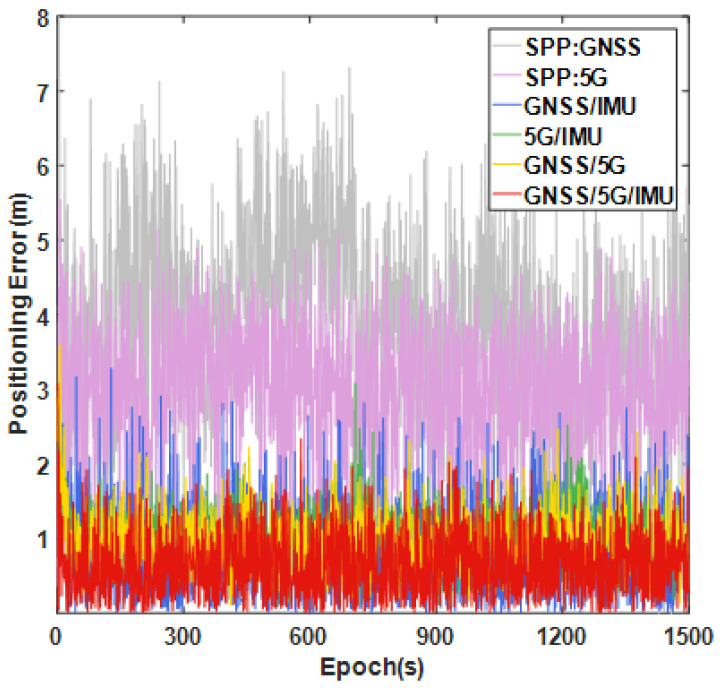
Comparison of fusion localization errors of different navigation sources.

To further verify the effectiveness of MCC-WRAF, this subsection evaluates the improvement of the proposed method and other fusion techniques for the positioning system in a fault-free scenario and compares these with positioning using a single data source. Figure 17 presents a comparison of the positioning errors across different navigation sources. The results demonstrate that compared with a single data source employing the weighted least squares method, the GNSS/IMU, 5G/IMU, GNSS/5G, and GNSS/5G/IMU fusion positioning algorithms all exhibited enhanced performance to varying degrees.

**Figure 18:**
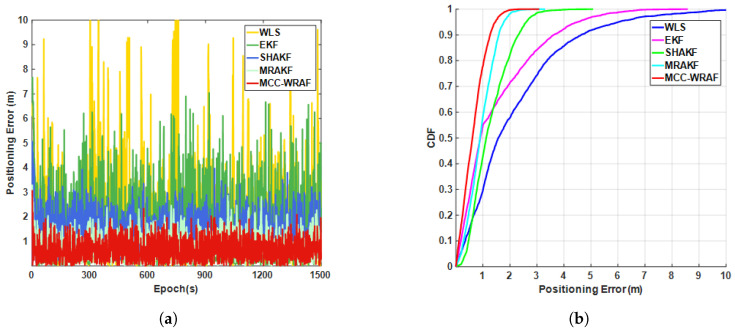
Fusion Methods for Failure-Free Scenarios: (**a**) Position Error and (**b**) CDF Curves.

Figure 18 shows a comparison of the positioning errors and CDFs for different fusion methods, while Table 4 provides a detailed comparison of the errors for various positioning methods, including the average error, standard deviation, RMSE, and (1σ) error. Among all fusion approaches, the WLS algorithm exhibited the poorest performance, with a (1σ) positioning error of 2.59 m. This is primarily because WLS relies solely on current measurements without accounting for temporal correlations or the system’s dynamic model, making it less adaptable to dynamic changes in real-world measurements. The EKF, by linearizing nonlinear equations using first-order Taylor expansion, considers temporal relationships. However, the fixed noise matrix in the system model limits its ability to handle dynamic observational changes, leading to limited robustness against outliers and, consequently, some loss of accuracy. During the operation of the algorithm, checks on the adaptive noise matrix revealed that it often failed to maintain a positive definite form, sometimes approaching singularity, indicating the limitations of the SHAKF algorithm. MRAKF, while requiring complex parameter tuning, demonstrated strengths in multi-rate processing capabilities and dynamic adaptability, effectively mitigating interference from low-frequency observations in high-frequency sensors. The proposed MCC-WRAF, employing a weighted robust adaptive filter based on maximum correntropy, exhibited the best performance. Compared with MRAKF, MCC-WRAF showed improvements of 29.3%, 12%, 24.7%, and 28.3% in its average error, standard deviation, RMSE, and 1σ error, respectively, which improved the dynamic localization accuracy of the fusion navigation system to 0.83 m (1σ), fully proving the superiority of the proposed method.

## 6. Discussion

The tightly coupled GNSS/5G/IMU framework, which incorporates robust weighted adaptive filtering and FDE functionality, is a crucial element to deliver high-integrity PNT services. Using massive MIMO, wide bandwidth, and millimeter-wave technologies, 5G provides positioning capabilities in challenging environments such as indoor areas and urban canyons, addressing GNSS limitations such as signal obstruction and frequent multipath or nonline-of-sight interference. The IMU serves as a reference source, maintaining functionality during brief signal outages to ensure system availability and reliability. Fault detection and exclusion in navigation systems are essential for defining the navigation and safety performance in multi-source fusion set-ups, reflecting the system’s ability to promptly alert users when it becomes unreliable.

We addressed full-source faults in a tightly coupled GNSS/5G/IMU navigation system, systematically analyzing the impact of undetected or residual faults on state estimation and proposing a fault detection and exclusion method based on filter innovation. The proposed multi-step fault separation accurately detected and differentiates GNSS, 5G, and IMU faults, and TSAIME was introduced to effectively and promptly identify faulty satellite or base station signals. Additionally, a fault exclusion strategy and sensor recovery verification method were devised to mitigate the effects of faults on system positioning performance, enabling the swift reintegration of reliable source sensors into the navigation solution. Moreover, a weighted robust adaptive filter based on maximum correntropy was designed to solve the non-positive definiteness problem of the covariance matrix during filter iteration, eliminating error propagation. Urban vehicle field experiments verified the effectiveness of the proposed method using typical fault scenarios. The results demonstrate that the method effectively distinguished GNSS, 5G, and IMU faults, eliminated error propagation, improved detection sensitivity under various fault conditions, shortened the alarm response times for ramp faults, and maintained the positioning performance during fault periods. The inclusion of the weighted robust adaptive filter also significantly reduced observation residual distortion during iterations, decreased the accumulation of errors, and improved the positioning accuracy. Meanwhile, this paper currently only gives empirical evidence and simulations for outdoor and urban canyon environments, and indoor deep occlusion scenarios are part of our future research program.

## 7. Conclusions

In this study, we focused on challenges in tightly coupled GNSS/5G/IMU positioning, including poor robustness, error tracking in innovation vectors, low FDE sensitivity, and insufficient usability of fusion results. To address these issues, we proposed a weighted robust adaptive filter based on the maximum correntropy criterion, along with fault detection and exclusion and sensor verification methods. The field experiments, combined with the simulation results, showed that the proposed method effectively reduced the negative effects of undetected faults, improved detection sensitivity, reduced alarm response times, and improved the dynamic positioning accuracy of the fusion navigation system to 0.83 m (1σ). This work will provide unprecedented accuracy and reliability of timing and positioning services for mass user terminals in complex occluded or semi-occluded positioning environments.

In future work, we will focus on analyzing the impact of the number and geometric distribution of satellites and 5G base stations on the positioning performance, analyzing and testing the complexity of multi-path and non-line-of-sight propagation in real environments, especially by conducting experiments in typical indoor scenarios, including further digging into visually assisted fault detection techniques in the case of severe obstruction of wireless signals and improving the robustness of the receiver’s multi-source fusion positioning, striving to provide reliable PNT services to the general public.

## Figures and Tables

**Table 1:** Key 5G parameter settings.

Parameters	Value
5G carrier frequency	3.5 GHz
5G bandwidth	100 MHz
Sub-carrier spacing	30 KHz
mMIMO antenna	UPA (12 × 12)
Total number of 5G BSs	10

**Table 2:** Detailed description of the added fault.

Case	Fault Source	Fault Time (s)	Fault Sensor	Fault Description
1	GNSS	1000–1040	SAT-17	15 m constant bias fault
2	GNSS/5G	1000–1040	SAT-17, BS-1	15 m constant bias fault
3	IMU	1000–1010	NONE	0.005 rad/s and 0.05 $m/s^{2}$ step faults per gyroscope and accelerometer axis
				0.05 rad/s and 0.5 $m/s^{2}$ step faults per gyroscope and accelerometer axis
				0.5 rad/s and 1 $m/s^{2}$ step faults per gyroscope and accelerometer axis
4	GNSS	980–1040	SAT-17	980–999 s add 5 m constant bias fault, 1000–1040 s add 15 m constant bias fault
5	GNSS	940–1040	SAT-13	0.1 m/s gradually increasing fault
6	GNSS/5G	940–1040	SAT-13, SAT-17, BS-2	0.1 m/s gradually increasing fault
7	GNSS	940–960	SAT-13, SAT-20	15 m constant bias fault
	GNSS/5G	980–1040	SAT-13, BS-2	0.2 m/s gradually increasing fault

**Table 3:** RMSEs of positioning during fault periods.

Case	RMSE with FDE in Different Direction (m)				RMSE Without FDE in Different Direction (m)			
	East	North	Up	All	East	North	Up	All
1	0.34	0.55	0.45	0.78	2.47	2.09	1.81	3.71
2	0.35	0.55	0.49	0.82	1.68	1.42	2.45	3.30
3.1	0.35	0.58	0.58	0.89	0.33	0.64	0.61	0.95
3.2	0.35	0.66	0.60	0.96	4.30	3.89	2.64	6.37
3.3	0.34	0.64	0.61	0.96	7.47	5.24	4.35	10.11
4 Stage 1	2.12	2.42	1.87	3.72	2.29	2.67	2.25	4.16
4 Stage 2	1.46	1.28	0.45	2.46	12.49	5.92	4.81	14.63
5	0.76	0.61	0.56	1.12	3.02	1.32	3.32	4.68
6	1.27	0.63	2.36	2.75	3.18	1.42	8.27	8.98
7 Stage 1	0.23	0.46	0.20	0.55	1.06	1.10	0.95	1.80
7 Stage 2	0.60	0.81	0.82	1.30	1.38	1.09	5.45	5.73

**Table 4:** Comparison of positioning performance of various fusion methods in fault-free scenarios.

Positioning Performance	Mean Error (m)	Standard Deviation (m)	Root Mean Square Error (m)	1σ (cdf68%) (m)
SPP:GNSS	3.81	1.29	4.03	4.40
SPP:5G	2.98	1.09	1.52	3.55
WLS	2.17	1.90	2.89	2.59
EKF	1.50	1.45	2.09	1.77
SHAKF	1.27	0.73	1.47	1.56
MRAKF	0.92	0.50	1.05	1.16
Proposed	0.65	0.44	0.79	0.83

## Data Availability

The data which support the findings of this study are available from the corresponding author upon reasonable request.
